# Impact of Masked Replacement of Sugar-Sweetened with Sugar-Free Beverages on Body Weight Increases with Initial BMI: Secondary Analysis of Data from an 18 Month Double–Blind Trial in Children

**DOI:** 10.1371/journal.pone.0159771

**Published:** 2016-07-22

**Authors:** Martijn B. Katan, Janne C. de Ruyter, Lothar D. J. Kuijper, Carson C. Chow, Kevin D. Hall, Margreet R. Olthof

**Affiliations:** 1 Department of Health Sciences, EMGO Institute for Health and Care Research, VU University, De Boelelaan 1085, 1081 HV Amsterdam, the Netherlands; 2 Laboratory of Biological Modeling, National Institute of Diabetes and Digestive and Kidney Diseases, Bethesda, MD, United States of America; Vanderbilt University, UNITED STATES

## Abstract

**Background:**

Substituting sugar-free for sugar-sweetened beverages reduces weight gain. This effect may be more pronounced in children with a high body mass index (BMI) because their sensing of kilocalories might be compromised. We investigated the impact of sugar-free versus sugary drinks separately in children with a higher and a lower initial BMI z score, and predicted caloric intakes and degree of compensation in the two groups.

**Methods and Findings:**

This is a secondary, explorative analysis of our double-blind randomized controlled trial (RCT) which showed that replacement of one 250-mL sugary drink per day by a sugar—free drink for 18 months significantly reduced weight gain. In the 477 children who completed the trial, mean initial weights were close to the Dutch average. Only 16% were overweight and 3% obese. Weight changes were expressed as BMI z-score, i.e. as standard deviations of the BMI distribution per age and sex group. We designated the 239 children with an initial BMI z-score below the median as ‘lower BMI’ and the 238 children above the median as ‘higher BMI’. The difference in caloric intake from experimental beverages between treatments was 86 kcal/day both in the lower and in the higher BMI group. We used a multiple linear regression and the coefficient of the interaction term (initial BMI group times treatment), indicated whether children with a lower BMI responded differently from children with a higher BMI. Statistical significance was defined as p ≤ 0.05. Relative to the sugar sweetened beverage, consumption of the sugar—free beverage for 18 months reduced the BMI z-score by 0.05 SD units within the lower BMI group and by 0.21 SD within the higher BMI group. Body weight gain was reduced by 0.62 kg in the lower BMI group and by 1.53 kg in the higher BMI group. Thus the treatment reduced the BMI z-score by 0.16 SD units more in the higher BMI group than in the lower BMI group (p = 0.04; 95% CI -0.31 to -0.01). The impact of the intervention on body weight gain differed by 0.90 kg between BMI groups (p = 0.09; 95% CI -1.95 to 0.14). In addition, we used a physiologically-based model of growth and energy balance to estimate the degree to which children had compensated for the covertly removed sugar kilocalories by increasing their intake of other foods. The model predicts that children with a lower BMI had compensated 65% (95% CI 28 to 102) of the covertly removed sugar kilocalories, whereas children with a higher BMI compensated only 13% (95% CI -37 to 63).

**Conclusions:**

The children with a BMI above the median might have a reduced tendency to compensate for changes in caloric intake. Differences in these subconscious compensatory mechanisms may be an important cause of differences in the tendency to gain weight. If further research bears this out, cutting down on the intake of sugar-sweetened drinks may benefit a large proportion of children, especially those who show a tendency to become overweight.

**Trial Registration:**

ClinicalTrials.gov NCT00893529

## Introduction

Randomized controlled trials suggest that sugar—free beverages reduce weight gain when compared with sugar—sweetened drinks [1,2]. In our double—blind trial in children we found that replacing one 250 mL (8 oz) can per day of sugary drink by an indistinguishable sugar-free beverage reduced weight gain by 1 kg in 18 months. Evidently the removal of liquid sugar is sensed incompletely by satiating mechanisms and is incompletely compensated for by additional consumption of other foods [3,4].

Our subjects mostly had a normal Body Mass Index (BMI). However, the effect of sugar-sweetened beverages may be more pronounced in obese children [5], because compensatory mechanisms may function less well in them [6,7]. This may work both ways: children may not notice that they are overeating, and they may also fail to perceive a fall in caloric intake. This suggestion has come up repeatedly in the literature but, as reviewed by Kaiser et al. [5], previous studies in lean versus overweight children on the effects of sugar-sweetened beverages were inconclusive. A retrospective observational study of Welsh et al. [8] noted that heavier children were twice as likely to remain or become overweight if they consumed sugar—sweetened beverages than their lean peers. An experiment by Ebbeling et al. [9] suggested that decreasing sugar-sweetened beverage consumption had a beneficial effect on body weight only in children in the upper tertile of BMI. Sichieri et al. [10] encouraged school classes to drink less sugar-sweetened beverages and found that BMI was significantly reduced only among overweight girls.

However, these and similar trials [11] were unblinded and could therefore not discriminate between conscious changes in behavior, and subconscious physiological mechanisms [12]. We recently reported the effect on body weight and fatness of substituting sugar—free for sugar—sweetened beverages in a double—blind trial in children aged 5-11. The double-blind design allowed the study of physiological mechanisms that were independent of behavioral cues and voluntary changes in consumption [1,13]. The hypothesis that the effect of sugar-sweetened beverages may depend on baseline BMI is intriguing and very relevant for future research as well as for public health. However, this hypothesis has not been formally investigated in blinded experimental studies. The data of our DRINK study data permit a first exploration of this hypothesis in a blinded setting and throughout the BMI spectrum and not only in obese children.

Here we report post-hoc analyses of the impact of sugar—free versus sugary drinks separately in children with an initial BMI z score below the median of the total group (‘lower BMI’) and children with a BMI above the median (‘higher BMI’). In addition, we used a previously developed and validated physiological model of growth and energy balance of children [14] to predict the degree of caloric compensation in the two groups. Some of our data were incorporated into the review of Kaiser et al.[5].

## Methods

### Design and study population

The Double-blind Randomized INtervention study in Kids (DRINK) was an 18-month double-blind randomized controlled trial in 641 children aged 5-11 living near Amsterdam, the Netherlands. The design and main outcomes of this trial are published [1,13]. This paper is a secondary analysis of 477 children who completed the trial. Our study was initially designed to test the effect of masked replacement of sugar-sweetened beverages with non-caloric beverages on weight gain in children. Here we explore the possibility of a differential BMI response to the treatment. For each child enrolled in the study, written informed consent was provided by a parent or guardian who had obtained assent from the child. The study protocol was approved by the Medical Ethics Committee of VU University Medical Center Amsterdam. Participants were individually randomized to receive 250 mL (8.45 oz) per day of either a sugar-free, artificially sweetened beverage with 0 kcal (sugar-free treatment) or a similar sugar-sweetened beverage that provided 104 kcal/d (sugar treatment). The aim of the study was to estimate the extent of spontaneous compensation for changes in the intake of liquid kilocalories. Our null hypothesis was that children who received the sugar-free treatment would fully compensate for the loss of the sugar calories from their drinks by increasing their intake of kilocalories from other foods and beverages. Therefore the children received no dietary or lifestyle instructions and they were free to follow their own dietary habits.

**Fig 1:**
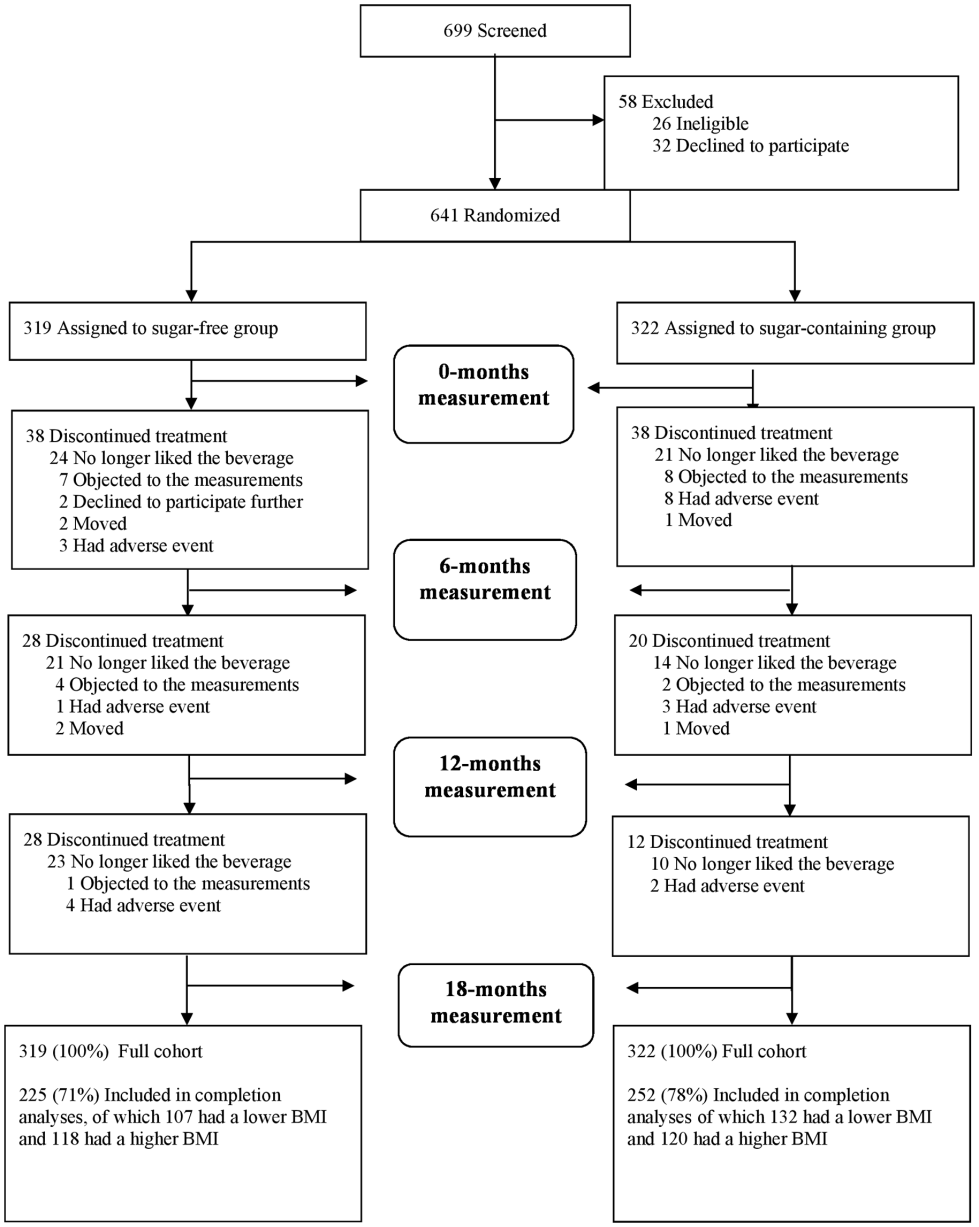
CONSORT flow diagram Screening, Randomization and Follow-up of the Participants.

We randomized the participants stratified by school (schools were within 35 km of each other), gender, age and initial BMI. Therefore the two treatment arms contained similar numbers of children with a higher BMI, and the same held for children with a lower BMI. Baseline characteristics were determined by questionnaire. Children were eligible only if they already drank sugar—sweetened beverages habitually during the morning break at school, because we considered it unethical to provide sugary beverages to children who did not habitually consume such beverages. Out of 641 children who started the study, 477 completed the 18 months intervention (Fig 1). Measurements made at 6 and 12 months and multiple imputation estimates suggested that the outcome of the trial was not affected substantially by differential dropout [1].

### Study beverages and measurements

Dutch primary school children habitually bring a snack and a beverage to school to be consumed during the morning break. We replaced the beverage brought from home with study beverages. We provided children with 1 can per day of a non-caloric, artificially sweetened, non-carbonated beverage or an equivalent sugar—sweetened non-carbonated beverage. The identical-looking and tasting 250-ml cans provided either 0 or 26 g of sucrose (0 or 104 kcal per day) [1,13]. We measured body weight, height, skinfold thickness of the biceps, triceps, and subscapular and suprailiac regions, waist circumference, and arm—to—leg electrical impedance, at 0, 6, 12, and 18 months [13].

### Classification of children into BMI group

**Fig 2:**
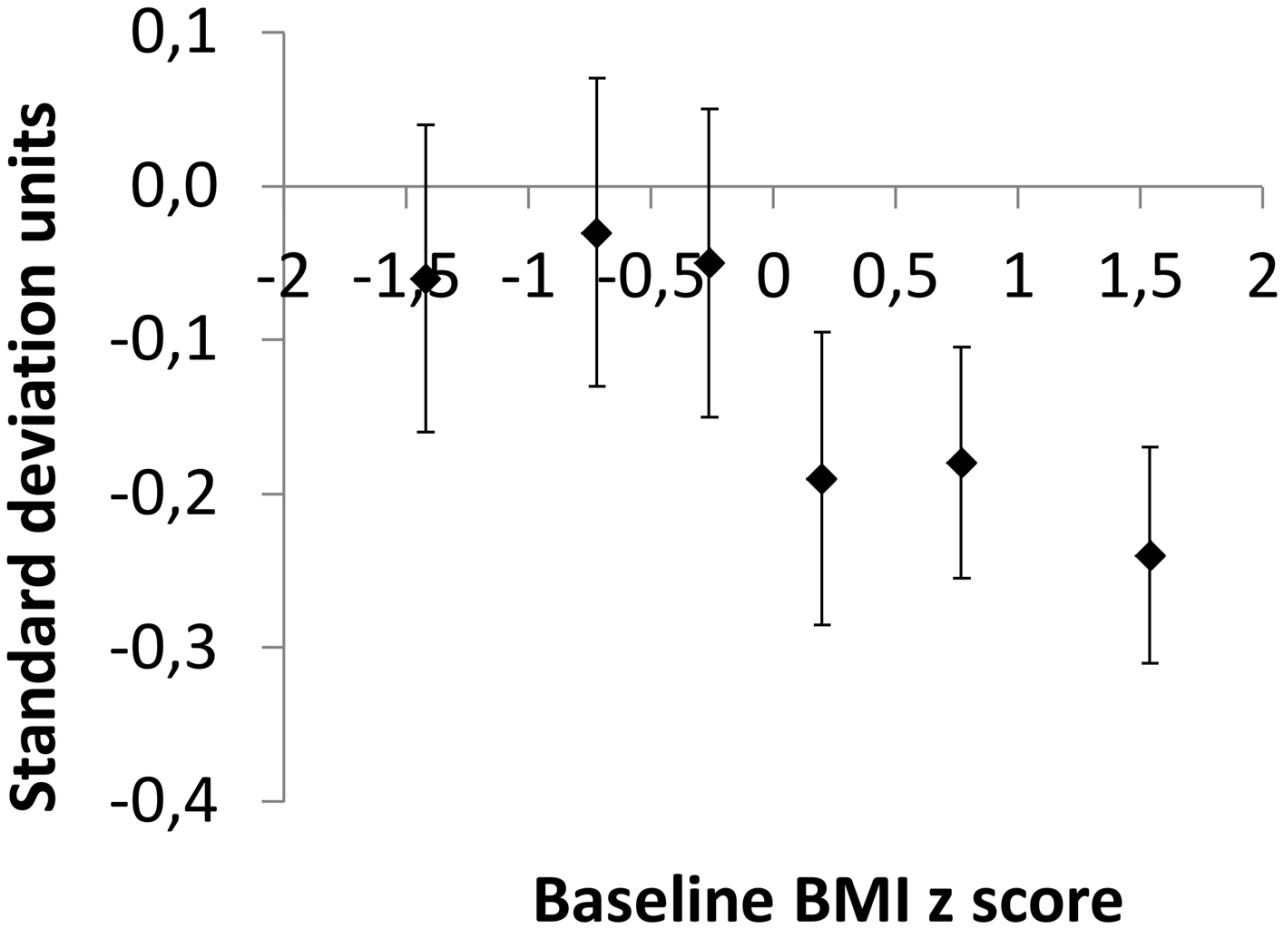
Effect of treatment (± SE) for 18 months with sugar free versus sugar sweetened beverages on BMI z score within six categories (sextiles) of initial BMI z score. The first sextile refers to the difference in change in BMI z score between 37 children with an initial BMI z score ≤ 1.03 who received sugar free and 43 children with an initial BMI z score ≤ 1.03 who received sugary drinks. Limits for the other sextiles are given in Methods.

The median initial BMI of the 477 children who completed the study was close to the nationwide median of Dutch children of the same age and gender [15]; the difference was -0.03 SD units. We classified the children into two groups: ‘lower BMI’, i.e. an initial z-score below the median of the study sample (N = 239; 132 of them received the sugar-free, and 107 the sugar-sweetened beverage), and ‘higher BMI’, i.e. initial z score above the median (N = 238; 120 received the sugar-free, and 118 the sugar-sweetened beverage. The internationally agreed cutoff points of Cole et al. [16,17] showed that 386 or 81% of the subjects had a low or healthy BMI at baseline and 91 or 19% were overweight or obese. In addition, the 477 children who completed the study were divided into sextiles, i.e. 6 equally sized categories, by their initial BMI (Fig 2). The BMI was expressed as a z-score, i.e. as the number of standard deviations by which the child’s BMI differed from the nationwide mean for his or her age and sex [15]. The six categories were: less than -1.03 BMI SD units below the median (N = 78), -1.03 to -0.46 (N = 79), -0.46 to -0.03 units (N = 82), -0.03 to 0.42 (N = 78), 0.42 to 1.10 units (N = 80), and more than 1.10 BMI SD units above the median (N = 80). Within each sextile, about half of the children had received sugary and the other half sugar-free beverages.

### Energy intake and caloric compensation

Since energy intake was not directly measured in this study, we used the physiologically-based child growth model of Hall et al. to predict caloric intakes and the degree of compensation in the lower and the higher BMI group. Details are in Hall et al, [14] and in S1 Appendix. The model has previously been shown to accurately account for growth in children. It is based on the physiology of growing children and has been validated with data from both healthy-weight and obese children. It accounts for growth of body fat and fat-free mass in terms of energy balance between intake and expenditure, including the energy cost of growth and the various organ contributions to resting metabolic rate. The model also accounts for the average age-related trends of physical activity and energy expenditure. An adult version of the model has been validated to accurately calculate free-living energy intake in 140 adults undergoing a 2 year calorie restriction experiment [18]. Because the model has been validated for children aged 6 years and over, the calculation of caloric compensation was limited to the 398 children aged 6–11 who completed the study. We first predicted the expected energy intake for each child according to gender, weight, and age at baseline and 18 months into the trial. We then calculated the mean change in energy intake by treatment and by BMI group over the 18 months of intervention. Finally, we calculated the increase in daily energy intake in the sugar treatment compared to the sugar-free treatment, again by initial BMI. This gave the predicted difference in caloric intake between treatments. The expected difference in intake from experimental beverages alone was 86 kcal/day after the mean compliance of 83% [1] had been taken into account. We calculated the average percentage compensation as 100 * (86—achieved difference)/86.

### Statistical analyses

We used a multiple linear regression model Z = a*X + b*Y+ c*X*Y + Ɛ, where Z is the change in BMI z score from 0 to 18 months, X is the initial BMI group (0 for lower BMI and 1 for higher BMI), and Y the treatment (0 for sugar-free and 1 for sugar-sweetened beverage). The lower case a, b, and c are the respective regression coefficients. This formal test for effect modification was also done in previous studies [2,9,19]. In a second model we also adjusted for gender, age, parental education, and parental ethnicity. The coefficient of the interaction term, for initial BMI group times Treatment, indicated whether children with a lower BMI responded differently from children with a higher BMI. We did similar analyses for body weight (kg), sum of skinfold thicknesses (mm), waist circumference (cm), electrical impedance fat mass (kg & %), and predicted energy intake. We repeated all analyses with initial BMI categorized into lean or overweight/obese [16,17] instead of below or above the median. In addition, we modeled the interaction more robustly by using BMI as a continuous variable in the regression model. The explorative character of the current study dictates that we interpret the statistical results with due restraint. However, it offers a unique opportunity to find potentially clinical relevant indications on effects of sugar sweetened beverages in specific groups that deserve future study. We used p≤0.05 as the limit for statistical significance of effect modification.

## Results

### Participants

The mean±SD baseline BMI z score of the 239 children with a lower BMI was -0.80±0.53 SD units relative to the Dutch median, and that of the 238 children with a higher BMI 0.83±0.60 SD units. Female gender, lower education of parents, and non-western ethnicity were somewhat overrepresented in the higher BMI group (Table 1). At baseline before the start of the trial, children with a lower BMI consumed a mean±SD of 1.03±0.22 sugary beverages at school during the morning break, and children with a higher BMI consumed 1.02±0.21 beverages, in agreement with school regulations that allow one drink in the morning break. In total 26% of children dropped out during the study; dropout rates were similar in the two treatment arms. While they were still in the study, changes in weight and body fat in future dropouts paralleled those in the children who completed the study. Minor adverse events were reported by 13% of participants [1].

**Table 1:** Baseline characteristics of participants who completed the Double-blind Randomized INtervention study in Kids. Participants were classified into two groups: ‘lower BMI’ (N = 239), i.e. an initial z score below the group median, and ‘higher BMI’ (N = 238), i.e. above the median^a^^,^^b^.

Characteristic	Lower BMI		Higher BMI	
	Sugar treatment (N = 132)	Sugar-free treatment (N = 107)	Sugar treatment (N = 120)	Sugar-free treatment (N = 118)
Girls (%)	36	41	50	47
Age (years)	8.2±1.9	8.0±1.8	8.1±1.8	8.2±1.9
Dutch ancestry (%)^c^	89	93	80	86
Non-western ancestry (%)	10	6	20	13
Low parental education (%)^d^	50	60	54	63
High parental education (%)	49	37	46	36
Weight (kg)	26.51±5.76	25.83±5.56	33.32±9.00	33.32±9.02
Height (cm)	131.7±12.2	130.2±11.7	133.2±12.9	133.1±13.0
Height z score (SD units relative to Dutch mean) ^e^	–0.17±0.99	–0.28±0.92	0.18±1.02	0.03±0.90
Body-mass index (kg/m^2^)	15.1±0.86	15.0±0.87	18.4±2.0	18.4±2.1
Body-mass index z score (SD units relative to Dutch mean) ^e^	–0.81±0.52	–0.80±0.54	0.85±0.61	0.82±0.59
Sum of four skinfolds (mm)	25.6±6.6	25.8±6.7	44.0±17.4	45.0±18.2
Waist-to-height ratio (%)	42.0±2.2	42.2±2.1	46.5±3.5	46.7±3.4
Electrical-impedance fat mass (kg)	3.8±1.9	3.8±1.7	7.3±3.4	7.3±3.8
Electrical-impedance fat mass (% of body weight)	14	14	21	21

^a^ Plus-minus values are means±SD

^b^ Group median for z-BMI was -0.03 SD units below Dutch mean [15]

^c^ A child was classified as Dutch if both parents were born in the Netherlands and as non-Western if one or both parents were born in Suriname, Dutch Antilles, Turkey, Morocco, Russia, Egypt, or Vietnam

^d^ We based the educational level on that of the parent or guardian who had the highest level of education

^e^ We calculated z score of body-mass index and height from the Dutch 2009 reference data [15].

### Compliance and blinding

The 477 children who completed the study consumed on average 5.8 cans or 83% of the assigned 7 cans per week, which resulted in an average achieved difference in caloric intake from treatment beverages of 86 kcal/d between treatments [1]. Children with a lower BMI consumed on average 5.7 cans per week, and children with a higher BMI 5.9 cans with minor differences between sugary and sugar-free beverages [1]. Blinding was by and large successful [1], but in both the higher and lower BMI groups the proportion of participants who were aware of the type of beverage that they had consumed was slightly higher in those who had received the sugar—free than in those who had received the sugar-sweetened beverage (Table D in S2 Appendix).

### BMI z score and other end points

The BMI z score increased less in children who received sugar-free, artificially sweetened drinks than in children who received sugar-sweetened beverages [1]. However, the difference between treatments depended markedly on initial BMI; when we divided children into sextiles of initial BMI z-score we noted a marked increase in the effect of treatment from the third to the fourth sextile, i.e. around the median BMI z score of Dutch children (Fig 2).

**Fig 3:**
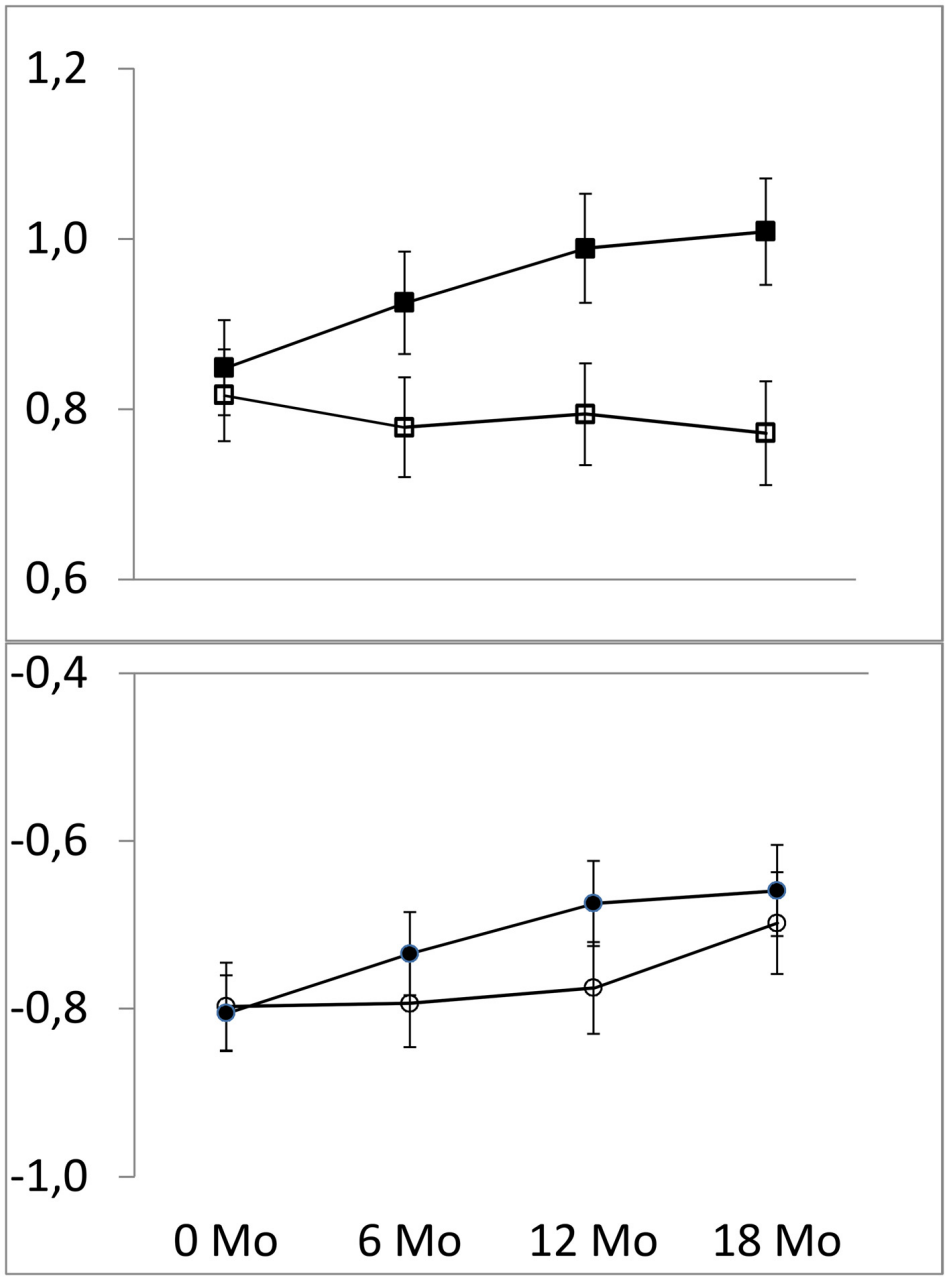
BMI z score (±SE) relative to the Dutch reference value in 239 children with an initial BMI z score below the study sample median (circles) and in 238 children with an initial BMI z score above the median (squares). Filled symbols denote children who received sugary beverages and open symbols children who received sugar free beverages.

Body fatness steadily declined over the course of the trial in children with a higher initial BMI who were unknowingly switched to sugar-free drinks (Fig 3, open squares), while masked removal of sugar had little impact on body fatness in the children with a lower initial BMI (Fig 3, open circles). In children who received sugary beverages, initial BMI had little impact on the course of BMI z score with time (Fig 3, closed symbols).

Consumption of the sugar-free beverage reduced the BMI z-score by 0.05 SD units within the lower BMI group and by 0.21 SD within the higher BMI group, both relative to the sugary beverage (Figs 2 and 3, Table 2 and Table A in S2 Appendix).

**Table 2:** Effect of sugary versus sugar-free beverages on measures of body fatness, by initial BMI. The data refer to the 477 children who remained in treatment for the full 18 months of the study ^a^.

Outcome	Initial BMI	Sugar-free treatment			Sugar treatment			Effect of treatment	Difference in effect of treatment between children with a lower vs. higher BMI^b^^c^	p-value	95%CI
		0 Mo	18 Mo	Change	0 Mo	18 Mo	Change				
BMI z score ^d^	Lower BMI	−0.80	−0.70	0.10	−0.81	−0.66	0.15	−0.05			
	Higher BMI	0.82	0.77	−0.04	0.85	1.01	0.16	−0.21	−0.16	0.04	−0.31 to −0.01
Body weight (kg)	Lower BMI	25.83	31.27	5.44	26.51	32.57	6.06	−0.62			
	Higher BMI	33.32	40.46	7.14	33.32	42.00	8.67	−1.53	−0.90	0.09	−1.95 to 0.14
Height (cm)	Lower BMI	130.22	140.24	10.02	131.71	142.11	10.40	−0.37			
	Higher BMI	133.08	143.46	10.38	133.16	143.93	10.77	−0.39	−0.02	0.96	−0.70 to 0.67
Sum of four skinfolds (mm)	Lower BMI	25.84	27.56	1.72	25.64	28.59	2.96	−1.24			
	Higher BMI	45.04	49.52	4.49	44.00	52.65	8.66	−4.17	−2.94	0.07	−6.16 to −0.29
Waist (cm)	Lower BMI	54.80	57.86	3.06	55.08	58.30	3.21	−0.16			
	Higher BMI	62.10	65.73	3.63	61.85	66.82	4.97	−1.34	−1.19	0.02	−2.21 to −0.16
Fat mass kg)	Lower BMI	3.78	4.38	0.58	3.82	4.73	0.92	−0.33			
	Higher BMI	7.26	8.68	1.42	7.26	9.52	2.31	−0.89	−0.55	0.10	−1.20 to 0.10
Fat mass (% body weight)	Lower BMI	14.37	13.60	−0.80	14.09	14.04	−0.05	−0.75			
	Higher BMI	20.70	20.27	−0.43	20.97	22.01	0.99	−1.42	−0.67	0.33	−2.03 to 0.69
Predicted energy intake (kcal/d)^e^	Lower BMI	1776	1945	169	1800	1999	199	-30			
	Higher BMI	2152	2357	206	2141	2422	280	-75	-45	0.10	-99 to 8

^a^ We classified the children as lower or higher BMI based on their initial BMI z score. ‘lower BMI’ refers to children with a BMI z score below the median of the study sample (sugar-free treatment N = 107; sugar treatment N = 132), and ‘higher BMI’ to children with a BMI z score above the median (sugar-free treatment N = 118; sugar treatment N = 120).

Values are means unless otherwise indicated. For standard deviations, see Table A in S2 Appendix.

^b^ unadjusted values. Outcomes were similar after adjustment for baseline characteristics, see Table A in S2 Appendix.

^c^ P≤0.05 was considered to indicate significant interaction between treatment and BMI group.

^d^ We calculated z scores of body-mass index and height from the Dutch 2009 reference data [15]

^e^ We estimated energy intakes with the model of Hall et al. [14] which has been validated for children aged 6 years and over. Therefore the prediction of caloric compensation was limited to the 398 children aged 6–11 who completed the study. Within the sugar free treatment 88 children had a lower and 97 children a higher initial BMI; within the sugar treatment 111 children had a lower and 102 children a higher initial BMI. P-values for the difference in estimated change in energy intake between treatments are: -75 kcal/d (P<0.001) for ‘‘higher BMI”, and -30 kcal/d (P value = 0.07) for ‘‘lower BMI”.

The impact of the treatment was thus 0.16 SD units (p = 0.04; 95% CI -0.31 to -0.01) larger in children with a higher than in children with a lower BMI (Fig 3, Table 2 and Table A in S2 Appendix). Children with a lower BMI who received sugar-free beverages gained 0.62 kg less weight than those in the sugar-sweetened treatment. The corresponding effect in children with a higher initial BMI was 1.53 kg. Thus the same treatment decreased weight gain by 0.90 kg more in children with a higher initial BMI than those with a lower BMI (p = 0.09; 95% CI -1.95 to 0.14). Similar differences in effect of treatment between BMI groups were seen for sum of skinfolds, waist circumference, and fat mass (Table 2 and Table A in S2 Appendix). When BMI is added as a continuous variable in the interaction term of the regression model the impact of treatment was -0.065 SD units (p = 0.086; 95% CI -0.14 to 0.009) per increase in BMI z-score at baseline (Table C in S2 Appendix). When we categorized initial BMI according to the international classification of Cole et al. [16,17], the treatment effect was 0.13 SD units larger in the 91 overweight and obese children than in the 386 children with a low or healthy initial BMI (p = 0.18; 95% CI -0.32 to 0.06) (Table B in S2 Appendix). Adjustments for gender, age, parental education and parental ethnicity did not affect the results (Table A in S2 Appendix).

### Energy intake and caloric compensation

The prediction of energy intake was limited to the 398 children aged 6–11 who completed the study. The relative weight loss was 0.9 kg in these children as compared with 1.0 kg in all 477 children [1] (Table E in S2 Appendix). The predicted mean increase in energy intake between the start of the trial and the finish 18 months later for children who received sugary drinks was 238 kcal/d, and 188 kcal/d for children who received sugar-free drinks. The difference in change in predicted energy intake between the treatments was therefore only 50 kcal per day (p<0.001), with the remaining 36 kcal/day, or 42%, being compensated by increased intake of other foods and drinks, or by reduced activity. We also estimated that if compensation had been zero, the deficit of 86 kcal/d would have led to a relative weight change difference of 1.9 kg in the low BMI and 2.0 kg in the high BMI children [14].

The degree of compensation differed between children with a lower and a higher initial BMI (p = 0.10, Table 2). The children in the lower BMI group achieved a difference in change in predicted total energy intake between the sugar treatment and the sugar-free treatment of 30 kcal/d (p = 0.07), with the remaining 56 kcal/d or 65% being compensated by increased intake of other foods and drinks. For the higher BMI group the achieved difference in change in estimated energy intake between treatments was 75 kcal/day (p<0.001); so these children compensated only 11 kcal/d or 13% by increased intake of other foods and drinks.

## Discussion

We earlier found that covert removal of sugar from children’s beverages reduced weight gain and body fat gain. The current analysis suggests that this effect may have been higher in children with a higher initial BMI. At first sight it seems puzzling that the effect of a fixed reduction in caloric intake of 86 kcal/d should lead to different effects on weight gain depending on initial BMI. The key to this effect is that children were unaware of whether they received sugar-free or sugary beverages, and were thus free to consume other foods and drinks as they liked. Our data showed that children with a lower initial BMI unconsciously compensated for most (65%) of the sugar removed from their beverages. In contrast, the children with a higher BMI who were randomized to receive sugar-free beverages appeared to replenish only 13% of the kilocalories removed from their morning drink. This led to a more pronounced reduction in weight gain in children with a higher initial BMI. Thus, an elevated BMI in children might predict a reduced tendency to compensate for changes in caloric intake and maintain body weight homeostasis. This had been suggested by earlier studies [6–10], but it had not been tested previously in a long-term double-blinded trial.

The impact of initial BMI on the degree of compensation was most evident in the trial arm in which children were switched to sugar-free beverages (Fig 3) and less evident in the sugary trial arm. This was to be expected because the sugary trial beverage was a continuation of what children had habitually brought to school for the morning break previous to the trial. It appears that the leaner children compensated more for the disappearance of sugar from their drinks by increased consumption more of other foods. They may also have decreased their activity, but evidence for this type of caloric compensation is scarce.

A unique feature of our trial was that it was double-blind. This makes it unlikely that children with a higher BMI consumed more of the sugary trial drinks than children with a lower BMI. This was confirmed by the empty cans count, which was similar in heavier and leaner children in both treatments. Thus, the most likely explanation of why the intervention had more impact in children with a higher baseline BMI is that their tendency to detect and compensate for masked removal of kilocalories is impaired. We speculate that children in the sugar-free treatment with a higher BMI failed to notice that kilocalories were missing from their morning drink, and they did not eat or drink extra to make up for this. It seems plausible that the effect works both ways, and that in an environment with plentiful kilocalories these children will tend to eat and drink more than they need [6,7]. The detrimental effect of initial BMI on the tendency to compensate was not limited to overweight or obese children, but was evident throughout a large part of the BMI range (Fig 2). Our data therefore suggest that a reduced tendency to detect and compensate for changes in liquid caloric intake might hold true also for children who are not yet overweight but who are only slightly heavier than their peers. There was a marked effect of the caloric manipulation on body fatness in each of the three sextiles of children with a baseline BMI z-score above the median (Fig 2). Out of the 50% of children above the median, 31% were lean by international criteria, 16% were overweight and only 3% met the criteria for obesity [16,17]. Therefore the fourth and most of the fifth sextile (Fig 2) consisted of children whose weight was within the ideal range, but they still appeared to be less able to compensate for the caloric manipulation. A prospective cohort study also found that children who were in the upper part of the normal weight distribution at age 5 were more likely to be obese by age 14 than those in the lower part of the normal weight distribution [20]. Apparently, a large proportion of children classified as having a normal weight are at risk to become overweight later in life, and even a slight plumpness may signal that a child is very vulnerable to the obesogenic environment created by modern society.

Even the thinnest children in our study were not immune to the effect of sugary drinks. The model of Hall et al. [14] allowed us to predict that in the heavier children, 87% of the kilocalories removed were not compensated for by changes in intake of other foods and drinks, whereas in kids with an initial BMI below the median 35% went uncompensated. An 8 oz. can of a sugary drink instead of a sugar-free drink still caused a weight difference of 0.6 kg in 18 months in these leaner children.

Our study had several strengths. The double—blind design made it unlikely that there was a conscious difference in food or drink intake between children with higher and lower BMI. Counting of returned cans suggested similar rates of adherence for children with a higher and lower BMI. Thus the effect was not caused by different intakes of study beverages but by the difference in caloric compensation as a physiological response to the removal of liquid kilocalories. Also, our sample size was larger than in previous studies [2,9,11], and the 18–month study duration ensured that the observed effect was not transient.

The difference of 0.16 SD units in change in BMI z score between children with a lower versus a higher BMI which we found is clinically relevant. The difference in BMI z-score we found in our study is in the same order of magnitude as the difference in BMI z-score between children whose parents had a low versus high education [15]. Previous studies have indicated that the prevalence of overweight is substantially higher in children whose parents had a low versus a high education. In a German study [21] the prevalence of overweight was 14.9% in children whose parents had a high education and 22,6% in children from parents with low education. Odds ratios for overweight (low vs high parental education) reached 3.1 in boys and 2.3 in girls. The effect we found in our study was achieved by a single small can (8 oz) per day of sugary drink, and children in the US consume almost 3 three times as many kilocalories from sugary drinks [22]. Therefore we speculate that decreased consumption of caloric beverages may reduce obesity prevalence in children.

Our study had certain limitations. First, the analysis of outcomes by baseline BMI was a post-hoc analysis, not pre-specified in the protocol. Second, confounding may be an issue, however multivariate adjustment left the effect of baseline BMI largely unchanged (Table A in S2 Appendix). Third, the proportion of participants who were aware of the type of beverage that they had consumed was slightly higher in those who had received the sugar—free than in those who had received the sugar-sweetened beverage. However, this proportion was the same in the higher and lower BMI groups (Table D in S2 Appendix). Finally, we were not able to assess actual dietary intakes of total energy and food components for logistic reasons.

The regulatory mechanisms underlying susceptibility to excess weight gain involve complex interactions among genetic predisposition [23], psychological factors, environmental stimuli [7,24,25], and also ethnic background may play a role [2]. Future studies will need to clarify whether children with a lower versus higher BMI indeed respond differently to the removal of liquid kilocalories, and the physiological mechanisms that explain such differences.

Our findings suggest that cutting down on the intake of sugar—sweetened drinks may benefit a large proportion of children, especially those who show a tendency to become overweight, but also in those for which overweight is not yet evident.

## Supporting Information

S1 Appendix Detailed description of the physiologically-based child growth model of Hall et al [14] (DOC)

S2 Appendix Supplementary tables A-E and full dataset of predicted energy intakes in table F. (DOCX)

S1 Checklist CONSORT Checklist. (DOC)

S1 Protocol Trial Protocol English version. (PDF)
