# Supplementary material for: Impact of Masked Replacement of Sugar-Sweetened with Sugar-Free Beverages on Body Weight Increases with Initial BMI: Secondary Analysis of Data from an 18 Month Double–Blind Trial in Children
Source: PLoS One. 2016 Jul 22;11(7):e0159771. doi: 10.1371/journal.pone.0159771 (PMC4957753; doi:10.1371/journal.pone.0159771)
Supplement: S1 Appendix — (DOC) [file pone.0159771.s001.doc]

**Supplementary Appendix 1**

Supplement to: Martijn B. Katan^1^ , Janne C. de Ruyter^1^, Lothar D.J. Kuijper^1^, Carson C. Chow^2^, Kevin D. Hall^2^, Margreet R. Olthof^1*^.

Impact of masked replacement of sugar-sweetened with sugar-free beverages on body weight increases with initial BMI: secondary analysis of data from an 18 month double–blind trial in children.

^1^Department of Health Sciences, EMGO Institute for Health and Care Research, VU University, De Boelelaan 1085, 1081 HV Amsterdam, the Netherlands.

^2^Laboratory of Biological Modeling, National Institute of Diabetes and Digestive and Kidney Diseases, Bethesda, MD 20895

*Corresponding author. E-mail: [margreet.olthof@vu.nl](mailto:margreet.olthof@vu.nl)

**Supplementary Materials**

**Impact of masked replacement of sugar-sweetened with sugar-free beverages on body weight increases with initial BMI: secondary analysis of data from an 18 month double–blind trial in children**

The description of the model is adapted from Reference 23.

Energy Partitioning

Our mathematical model of energy partitioning during childhood is an extension of our previously published adult model ^1-3^ and is described by the following equations:

$\begin{matrix} \frac{d\left(\rho_{FFM}FFM\right)}{dt}=p\left(I-E\right)+g\left(t\right) \\ \rho_{FM}\frac{dFM}{dt}=\left(1-p\right)\left(I-E\right)-g\left(t\right) \end{matrix}$ [1]

where the additional term, *g*(*t*), is a simplified model of growth as a function of age alone and represents the net effect of a variety of complex physiological processes that stimulate accretion of fat-free mass with an increased utilization of fat. For example, growth hormone is known to increase body protein synthesis as well as stimulate adipose lipolysis and therefore has the net effect on energy partitioning indicated in equation 1. Equation (1) is structured to implicitly obey macronutrient and energy balance.

The energy density of fat-free mass, ρ_FFM_, increases with age primarily because young children have a relatively greater hydration of fat-free mass ^4^. To account for this, we expand the rate of energy storage in fat-free mass as follows:

$\frac{d\left(\rho_{FFM}FFM\right)}{dt}=FFM\frac{d\rho_{FFM}}{dt}+\rho_{FFM}\frac{dFFM}{dt}\equiv\hat{\rho}_{FFM}\frac{dFFM}{dt}$ [2]

where we have defined the effective energy density of fat-free mass changes, $\hat{\rho}_{FFM}$, that is calculated as:

$\hat{\rho}_{FFM}=\rho_{FFM}+FFM\frac{d\rho_{FFM}}{dFFM}$ [3]

Assuming that the metabolizable energy content of fat-free mass is primarily due to body protein with an energy density of 4.7 kcal/g, the reference data of Fomon ^5^ and Haschke ^6^ was used to determine the linear relationship $\hat{\rho}_{FFM}\approx4.3\times FFM+837$ kcal/kg as shown in Figure S1.

Therefore, the energy partitioning model can be written as follows:

$\begin{matrix} \hat{\rho}_{FFM}\frac{dFFM}{dt}=p\left(I-E\right)+g\left(t\right) \\ \rho_{FM}\frac{dFM}{dt}=\left(1-p\right)\left(I-E\right)-g\left(t\right) \end{matrix}$ [4]

The energy partitioning p-ratio is given by the Forbes relationship ^1, 7, 8^:

$p=\frac{C}{C+FM}$ [5]

where

$C=10.4\text{ kg}\times\left(\frac{\hat{\rho}_{FFM}}{\rho_{FM}}\right)$. [6]

While we have used the adult Forbes relationship to define the energy partitioning p-ratio in equations 5 and 6, the presence of a non-zero growth term, g(t), in equation 1 means that energy partitioning does not follow the adult Forbes model during growth.

Energy Balance and Growth

Summing the energy partitioning equations 4 gives the energy balance equation:

$I-E=\hat{\rho}_{FFM}\frac{dFFM}{dt}+\rho_{FM}\frac{dFM}{dt}$ [7]

During normal growth, both body fat and fat-free mass increase with age at varying rates. Using the reference body composition data for males and females, *FFM_ref_* and *FM_ref_*, equation 7 allows us to calculate the reference energy imbalance gap, $EB_{ref}=\left(I-E\right)$, during normal growth in males and females. The reference body composition time course also allows us to solve equation 4 for the required growth function to match the reference body composition data:

[8]

If the reference body composition data during growth follow the adult Forbes relationship, the growth function *g*(*t*) = 0.

By fitting to data in References 5 and 6, we obtained the following time dependent functions:

[9a]

for girls, and

[9b]

for boys.

Energy Expenditure

We previously presented a mathematical framework for modeling energy expenditure during tissue deposition and showed that the energy expenditure equation during growth can be harmonized with adult models.^9^ Therefore, our model of childhood energy expenditure rate, E, was modeled using the same general equation as the adult model ^2, 3^:

$E=K+\gamma_{FFM}FFM+\gamma_{FM}FM+\beta\Delta I+\delta BW+\eta_{FFM}\frac{dFFM}{dt}+\eta_{FM}\frac{dFM}{dt}$ [10]

where *K* is a constant determined by the initial energy balance condition, η_FM_ = 180 kcal/kg and η_FFM_ = 230 kcal/kg account for the biochemical efficiencies associated with fat and protein synthesis ^9^ assuming that the change of fat-free mass are primarily accounted for by body protein and its associated intracellular water ^10^. The parameter *β* = 0.24 accounts for adaptation of energy expenditure during a diet perturbation Δ*I* away from the reference energy intake associated with normal growth ^9, 11^as calculated by:

[11]

The specific metabolic rate of fat tissue was$\gamma_{FM}$= 4.5 kcal/kg/d ^12^. Because the relative proportion of fat-free mass due to high metabolic rate organs is much higher in childhood ^12-14^, we calculated$\gamma_{FFM}$using the specific metabolic rates of the organs multiplied by the rate of organ growth with respect to fat-free mass accretion according to the following equation:

$\gamma_{FFM}=\sum_{i} \gamma_{i}\frac{dM_{i}}{dFFM}$ [12]

where *γ_i_* and *M_i_* are the average specific metabolic rate and mass of the organ indexed by *i*, respectively. The organs included liver (*γ_L_* = 200 kcal/kg/d, $dM_{L}/dFFM$=0.0256), kidney (*γ_K_* = 440 kcal/kg/d, $dM_{K}/dFFM$=0.0044), heart (*γ_H_* = 440 kcal/kg/d, $dM_{H}/dFFM$=0.0058), and residual lean tissue mass (*γ_R_* = 12 kcal/kg/d, $dM_{R}/dFFM$=0.96) as provided by Elia ^12^ and the organ growth rates were determined using data from Altman and Dittmer ^15^.

While the resting metabolic rate per unit bodyweight is known to be significantly higher in children due to the relative increased contribution of high metabolic rate organs^12-14^, we found that accounting for normal organ growth with fat-free mass accretion during childhood resulted in a calculated value of $\gamma_{FFM}$= 22 kcal/kg/d which is almost identical to the value used in our adult model ^2, 3^ . Thus, the predicted change in resting metabolic rate during the accretion of fat-free mass in childhood is in line with expectations from the adult model parameter values.

Physical activity expenditure was assumed to be proportional to bodyweight and was represented by δ in equation 10. The physical activity parameter δ was assumed to be a decreasing function of age ^18-21^:

$\delta(t)=\delta_{\min}+\frac{\left(\delta_{\max}-\delta_{\min}\right)P^{h}}{t^{h}+P^{h}}$ [13]

The parameter δ_min_ = 10 kcal/kg/d was chosen to correspond to an average young adult. To match the estimated physical activity levels in males and females as a function of age, we chose the mid point of the decline in physical activity at P = 12 years and the rate of decline was set using a Hill coefficient h = 12. The maximum physical activity, δ_max_, was chosen to be 19 kcal/kg/d and 17 kcal/kg/d in males and females, respectively.

The closed form expression for the energy expenditure rate is given by:

[14]

Predicting Food Intake

For the model time dependent functions (9), the model fat mass and fat free mass will grow according to an average reference child for each sex. We account for individual differences by adding or subtracting a fixed amount of energy excess or deficit per day. The predicted food intake for each child is then estimated by using Newton’s method with empirical derivatives to optimize the food intake such that the body composition matches the data at the target age. The initial intakes are estimated by assuming that the children have the same body composition at age 5 and then the model is time advanced to the age at the start of the study and matched to the body weight data.

**References**

1. Chow CC, Hall KD. The dynamics of human body weight change. PLoS Computational Biology. 2008; **4**(3): e1000045.

2. Hall KD, Guo J, Dore M, Chow CC. The progressive increase of food waste in America and its environmental impact. PLoS One. 2009; **4**(11): e7940.

3. Hall KD, Sacks G, Chandramohan D, Chow CC, Wang YC, Gortmaker SL, et al. Quantification of the effect of energy imbalance on bodyweight. Lancet. 2011; **378**(9793): 826-37.

4. Forbes GB. Human body composition: Growth, aging, nutrition, and activity. New York: Springer-Verlag; 1987.

5. Fomon SJ, Haschke F, Ziegler EE, Nelson SE. Body composition of reference children from birth to age 10 years. Am J Clin Nutr. 1982; **35**(5 Suppl): 1169-75.

6. Hashke F. Body composition during adolescence. In: Klish WJ, Kretchmer N, editors. Body composition measurements in infants and children. Columbus, OH: Ross Laboratories; 1989. p. 76-83.

7. Forbes GB. Lean body mass-body fat interrelationships in humans. Nutr Rev. 1987; **45**(8): 225-31.

8. Hall KD. Body fat and fat-free mass inter-relationships: Forbes's theory revisited. Br J Nutr. 2007; **97**(6): 1059-63.

9. Hall KD. Mathematical modelling of energy expenditure during tissue deposition. Br J Nutr. 2010; **104**(1): 4-7.

10. Hall KD. What is the required energy deficit per unit weight loss? Int J Obes (Lond). 2008; **32**(3): 573-6.

11. Hall KD, Jordan PN. Modeling weight-loss maintenance to help prevent body weight regain. Am J Clin Nutr. 2008; **88**(6): 1495-503.

12. Elia M. Organ and tissue contribution to metabolic rate. In: Kinney JM, Tucker HN, editors. Energy Metabolism: Tissue Determinants and Cellular Corollaries. New York: Raven Press; 1992. p. 61-79.

13. Holliday MA. Metabolic rate and organ size during growth from infancy to maturity and during late gastation and early infancy. Pediatrics. 1971; **47**(1): Suppl 2:169+.

14. Wang Z. High ratio of resting energy expenditure to body mass in childhood and adolescence: a mechanistic model. Am J Hum Biol. 2012; **24**(4): 460-7.

15. Altman PL, Dittmer DS. Growth, including reproduction and morphological development. Washington D.C.: Federation of American Societies for Experimental Biology; 1962.

16. Talbot FB. Basal metabolism standards for children. Am J Dis Child. 1938; **55**: 455-9.

17. Schofield WN. Predicting basal metabolic rate, new standards and review of previous work. Hum Nutr Clin Nutr. 1985; **39C**: 5-41.

18. Gortmaker SL, Lee R, Cradock AL, Sobol AM, Duncan DT, Wang YC. Disparities in youth physical activity in the United States: 2003-2006. Med Sci Sports Exerc. 2012; **44**(5): 888-93.

19. Sallis JF. Age-related decline in physical activity: a synthesis of human and animal studies. Med Sci Sports Exerc. 2000; **32**(9): 1598-600.

20. Caspersen CJ, Pereira MA, Curran KM. Changes in physical activity patterns in the United States, by sex and cross-sectional age. Med Sci Sports Exerc. 2000; **32**(9): 1601-9.

21. Troiano RP, Berrigan D, Dodd KW, Masse LC, Tilert T, McDowell M. Physical activity in the United States measured by accelerometer. Med Sci Sports Exerc. 2008; **40**(1): 181-8.

22. Swinburn BA, Jolley D, Kremer PJ, Salbe AD, Ravussin E. Estimating the effects of energy imbalance on changes in body weight in children. Am J Clin Nutr. 2006; **83**(4): 859-63.

23. Hall, KD, Butte, NF, Swinburn, BA, Chow, CC. Dynamics of childhood growth and obesity: development and validation of a quantitative mathematical model. *The Lancet Diabetes & Endocrinology* **1,** 97–105 (2013).
