# Supplementary material for: Impact of Masked Replacement of Sugar-Sweetened with Sugar-Free Beverages on Body Weight Increases with Initial BMI: Secondary Analysis of Data from an 18 Month Double–Blind Trial in Children
Source: PLoS One. 2016 Jul 22;11(7):e0159771. doi: 10.1371/journal.pone.0159771 (PMC4957753; doi:10.1371/journal.pone.0159771)
Supplement: S2 Appendix — (DOCX) [file pone.0159771.s002.docx]

**Supplementary Appendix 2**

Supplement to: Martijn B. Katan^1^ , Janne C. de Ruyter^1^, Lothar D.J. Kuijper^1^, Carson C. Chow^2^, Kevin D. Hall^2^, Margreet R. Olthof^1*^.

Impact of masked replacement of sugar-sweetened with sugar-free beverages on body weight increases with initial BMI: secondary analysis of data from an 18 month double–blind trial in children.

^1^Department of Health Sciences, EMGO Institute for Health and Care Research, VU University, De Boelelaan 1085, 1081 HV Amsterdam, the Netherlands.

^2^Laboratory of Biological Modeling, National Institute of Diabetes and Digestive and Kidney Diseases, Bethesda, MD 20895

*Corresponding author. E-mail: [margreet.olthof@vu.nl](mailto:margreet.olthof@vu.nl)

**Contents:**

**Page 3:** **Table A.** Study outcomes of the Double–blind Randomized INtervention study in Kids by baseline BMI. The data refer to the 477 children who remained in treatment for the full 18 months of the study. The B refers to the difference in change from baseline in children with a lower versus higher BMI.

**Page 5: Table B.** Study outcomes of the Double–blind Randomized INtervention study in Kids by baseline BMI. The data refer to the 477 children who remained in treatment for the full 18 months of the study. The B refers to the difference in change from baseline in lean versus overweight/obese children.

**Page 7: Table C.** Study outcomes of the Double–blind Randomized INtervention study in Kids by baseline BMI. The data refer to the 477 children who remained in treatment for the full 18 months of the study when baseline BMI z score is included as continuous term instead of in categories.

**Page 8: Table D.** Responses of participants by baseline BMI who finished the trial to the question which type of beverage they thought they had consumed, by baseline BMI.

**Page 9: Table E.** Body weight (mean ± SD) of the 398 children aged 6-11 y for whom the model of Hall et al.[1] has been validated. All children completed the Double–blind Randomized INtervention study in Kids. They were classified as having a lower (n=199) or a higher initial BMI (n=199).

**Page 10:** **Table F**. The full dataset of predicted energy intakes of the 398 children aged 6-11 y for whom the model of Hall et al.[1] has been validated.

| **Table A.** Study outcomes of the Double–blind Randomized INtervention study in Kids by baseline BMI.^a^ The data refer to the 477 children who remained in treatment for the full 18 months of the study. The B refers to the difference in change from baseline in children with a lower versus higher BMI. | | | | | | | | | | | | | | | |
| --- | --- | --- | --- | --- | --- | --- | --- | --- | --- | --- | --- | --- | --- | --- | --- |
|  | **Outcome** | **Sugar–free treatment** | | | **Sugar treatment** | | | **Effect of treat-ment**^b^ | **95% CI** | **Difference in effect of treatment between children with a lower vs. higher BMI**^cd^ | **p-value** | **95% CI** | **Difference in effect of treatment between children with a lower vs. higher BMI**^cd^ | **p-value** | **95% CI** |
|  |  | 0 mo | 18 mo | Change | 0 mo | 18 mo | Change |  |  | B  Model 1^cd^ |  |  | B  Model 2^cd^ |  |  |
| Lower BMI^a^ | BMI z score ^e^ | –0.8±0.5 | ‑0.7±0.6 | 0.1±0.5 | –0.8±0.5 | –0.7±0.6 | 0.2±0.5 | –0.05 | -0.16 to 0.07 | Ref | Ref | Ref | Ref | Ref | Ref |
| Higher BMI | BMI z score ^e^ | 0.8±0.6 | 0.8±0.7 | –0.04±0.3 | 0.9±0.6 | 1.0±0.7 | 0.2±0.4 | –0.21 | -0.30 to -0.11 | –0.16 | 0.04 | –0.31 to –0.01 | –0.16 | 0.04 | –0.30 to –0.01 |
| Lower BMI | Body weight (kg) | 25.8±5.6 | 31.3±6.7 | 5.4±2.2 | 26.5±5.8 | 32.6±7.9 | 6.1±2.7 | –0.62 | -1.26 to -0.01 | Ref | Ref | Ref | Ref | Ref | Ref |
| Higher BMI | Body weight (kg) | 33.3±9.0 | 40.5±10.8 | 7.1±2.9 | 33.3±9.0 | 42.0±11.2 | 8.7±3.6 | –1.53 | -2.35 to -0.70 | –0.90 | 0.09 | –1.95 to 0.14 | –0.96 | 0.05 | –1.90 to –0.01 |
| Lower BMI | Height (cm) | 130.2±11.7 | 140.2±11.6 | 10.0±1.9 | 131.7±12.2 | 142.1±12.5 | 10.4±1.8 | –0.37 | -0.84 to 0.09 | Ref | Ref | Ref | Ref | Ref | Ref |
| Higher BMI | Height (cm) | 133.1±13.0 | 143.5±12.7 | 10.38±1.84 | 133.2±12.9 | 143.9±13.3 | 10.8±2.1 | –0.39 | -0.89 to 0.11 | –0.02 | 0.96 | –0.70 to 0.67 | 0.14 | 0.68 | –0.53 to 0.81 |
| Lower BMI | Sum of four skinfolds (mm) | 25.8±6.7 | 27.6±8.6 | 1.72±5.32 | 25.6±6.6 | 28.6±9.7 | 3.0±6.7 | –1.24 | -2.81 to 0.34 | Ref | Ref | Ref | Ref | Ref | Ref |
| Higher BMI | Sum of four skinfolds (mm) | 45.0±18.2 | 49.5±22.0 | 4.5±9.9 | 44.0±17.4 | 52.7±21.7 | 8.7±12.1 | –4.17 | -6.99 to -1.35 | –2.94 | 0.07 | –6.16 to 0.29 | –2.82 | 0.09 | –6.09 to 0.45 |
| Lower BMI | Waist circumference (cm) | 54.8±4.1 | 57.9±4.6 | 3.1±2.2 | 55.1±3.6 | 58.3±4.6 | 3.2±2.4 | –0.16 | -0.74 to 0.43 | Ref | Ref | Ref | Ref | Ref | Ref |
| Higher BMI | Waist circumference (cm) | 62.1±7.1 | 65.7±8.2 | 3.6±3.1 | 61.9±7.0 | 66.8±7.7 | 5.0±3.5 | –1.34 | -2.19 to -0.50 | –1.19 | 0.02 | –2.21 to –0.16 | –1.17 | 0.03 | –2.20 to –0.14 |
| Lower BMI | Fat mass on electrical impedance (kg) | 3.8±1.7 | 4.4±2.2 | 0.6±1.4 | 3.8±1.9 | 4.7±2.6 | 0.9±1.4 | –0.33 | -0.69 to 0.03 | Ref | Ref | Ref | Ref | Ref | Ref |
| Higher BMI | Fat mass on electrical impedance (kg) | 7.3±3.8 | 8.7±4.7 | 1.4±1.8 | 7.3±3.4 | 9.5±4.6 | 2.3±2.4 | –0.89 | -1.43 to -0.34 | –0.55 | 0.10 | –1.20 to 0.10 | –0.50 | 0.13 | –1.15 to 0.14 |
| Lower BMI | Fat mass on electrical impedance as %  of body weight | 14.4±4.6 | 13.6±5.1 | –0.8±3.8 | 14.1±4.9 | 14.0±5.4 | –0.1±3.6 | –0.75 | -1.70 to 0.19 | Ref | Ref | Ref | Ref | Ref | Ref |
| Higher BMI | Fat mass on electrical impedance as %  of body weight | 20.7±6.8 | 20.3±7.7 | –0.4±3.6 | 21.0±5.9 | 22.0±6.9 | 1.0±4.0 | –1.42 | -2.41 to -0.44 | –0.67 | 0.33 | –2.03 to 0.69 | –0.60 | 0.39 | –1.95 to 0.76 |
| Lower BMI | Energy intake (kcal) ^f^ | 1776±209 | 1945±224 | 169±113 | 1800±193 | 1999±242 | 199±114 | -30 | -62 to 2 | Ref | Ref | Ref | Ref | Ref | Ref |
| Higher BMI | Energy intake (kcal) | 2152±318 | 2357±331 | 206±136 | 2141±313 | 2422±375 | 281±171 | -75 | -118 to -32 | -45 | 0.10 | -98 to 8 | -39 | 0.16 | -93 to 16 |
| ^a^ We divided the children in lower versus higher BMI based on median of the initial BMI z score. Children with a lower BMI refers to children with a BMI z score ≤ –0.03 (sugar‑free treatment N=107; sugar treatment N=132), children with a higher BMI refers to children with a BMI z score > –0.03 (sugar‑free treatment N=118; sugar treatment N = 120). Values are mean±SD, unless otherwise indicated  ^b^ We examined the difference in study outcome between children with a lower or higher BMI by linear regression using SPSS 20.0. Values are  regression coefficients. The B refers to the differences in changes from baseline in the sugar‑free treatment relative to sugar treatment  ^c^ Model 1 was the crude model, Model 2 was adjusted for age, gender, parental education (lower to high school education, or college/ university education) and parental ethnicity ((Dutch, or non–western).  ^d^ Values are regression coefficients (p-value). The B refers to the change from baseline in children with a higher BMI relative to children with a lower BMI, and indicates the interaction term that multiplied treatment (0 or 1) with the initial BMI (0 or 1); P≤0.05 was considered to indicate significance.  ^e^ We calculated z score of body–mass index and height from the Dutch 2009 reference data [[1](#_ENREF_1)]  ^f^ Since the predictions for energy intake were only possible in children 6 years and over, we could only include 199 children in the lower BMI group and 199 in the higher BMI group. | | | | | | | | | | | | | | | |

| **Table B.** Study outcomes of the Double–blind Randomized INtervention study in Kids by baseline BMI. ^a^  The data refer to the 477 children who remained in treatment for the full 18 months of the study. The B refers to the difference in change from baseline in lean versus overweight/obese children. | | | | | | | | | | | | | | | | | | | |
| --- | --- | --- | --- | --- | --- | --- | --- | --- | --- | --- | --- | --- | --- | --- | --- | --- | --- | --- | --- |
|  | **Outcome** | **Sugar‑free treatment** | | | | **Sugar treatment** | | | | **Effect of treatment**^b^ | **95% CI** | **Difference in effect of treatment between children with a lower vs. higher BMI^cd^** | **p-value** | **95% CI** | **Difference in effect of treatment between children with a lower vs. higher BMI^cd^** | | **p-ue p-value** | **95%CI** |  |
|  |  | 0 mo | 18 mo | Change | | 0 mo | | 18 mo | Change |  |  | B  Model 1^cd^ |  |  | B  Model 2^cd^ | |  |  |  |
| Lean^a^ | BMI z score ^e^ | –0.3±0.  7 | –0.3±0.8 | 0.1±0.4 | –0.4±0.8 | | –0.2±0.9 | | 0.2±0.4 | –0.10 | –0.19 to –0.02 | Ref | Ref | Ref | Ref | | Ref | Ref |  |
| Overweight | BMI z score ^e^ | 1.4±0.4 | 1.3±0.5 | –0.1±0.3 | 1.5±0.4 | | 1.7±0.4 | | 0.2±0.3 | –0.23 | –0.36 to –0.10 | –0.13 | 0.17 | –0.32 to 0.06 | –0.13 | | 0.18 | –0.32 to 0.06 |  |
| Lean | Body weight (kg) | 27.6±6.7 | 33.5±8.3 | 5.9±2.5 | 28.1±7.0 | | 34.7±9.1 | | 6.6±3.0 | –0.72 | –1.27 to –0.17 | Ref | Ref | Ref | Ref | | Ref | Ref |  |
| Overweight | Body weight (kg) | 38.2±9.2 | 46.2±10.7 | 7.9±3.0 | 37.6±9.0 | | 48.0±10.7 | | 10.3±3.7 | –2.43 | –3.82 to –1.04 | –1.71 | 0.01 | –3.03 to –0.40 | –2.16 | | 0.00 | –3.35 to –0.97 |  |
| Lean | Height (cm) | 130.6±12.2 | 140.8±12.1 | 10.2±1.9 | 131.8±12.8 | | 142.3±13.1 | | 10.5±2.0 | –0.25 | –0.64 to 0.13 | Ref | Ref | Ref | Ref | | Ref | Ref |  |
| Overweight | Height (cm) | 136.1±12.4 | 146.3±11.9 | 10.2±1.7 | 135.3±11.1 | | 146.3±11.7 | | 11.0±1.8 | –0.87 | –1.60 to –0.14 | –0.62 | 0.16 | –1.48 to 0.25 | –0.66 | | 0.13 | –1.51 to 0.19 |  |
| Lean | Sum of four skinfolds (mm) | 29.9±10.4 | 32.3±13.5 | 2.4±7.0 | 29.6±9.6 | | 34.1±14.6 | | 4.5±8.7 | –2.07 | –3.67 to –0.48 | Ref | Ref | Ref | Ref | Ref | | Ref |  |
| Overweight | Sum of four skinfolds (mm) | 59.5±16.9 | 65.7±20.2 | 6.2±11.2 | 56.3±19.9 | | 67.5±20.1 | | 11.2±13.6 | –4.99 | –10.18 to 0.20 | –2.91 | 0.16 | –7.02 to 1.19 | –2.87 | 0.18 | | –7.02 to 1.28 |  |
| Lean | Waist circumference (cm) | 56.5±5.0 | 59.7±5.9 | 3.2±2.4 | 56.5±4.7 | | 60.2±5.8 | | 3.7±2.8 | –0.48 | –1.01 to 0.05 | Ref | Ref | Ref | Ref | Ref | | Ref |  |
| Overweight | Waist circumference (cm) | 67.0±6.8 | 70.9±7.9 | 3.9±3.6 | 66.6±6.9 | | 72.3±7.0 | | 5.6±3.7 | –1.75 | –3.28 to –0.22 | –1.27 | 0.06 | –2.58 to 0.05 | –1.41 | 0.04 | | –2.72 to –0.09 |  |
| Lean | Fat mass on electrical impedance (kg) | 4.4±2.2 | 5.3±3.0 | 0.8±1.6 | 4.6±2.4 | | 5.8±3.3 | | 1.2±1.8 | –0.39 | –0.73 to –0.06 | Ref | Ref | Ref | Ref | Ref | | Ref |  |
| Overweight | Fat mass on electrical impedance (kg) | 10.2±3.5 | 12.0±4.5 | 1.9±1.9 | 9.4±3.6 | | 12.7±4.5 | | 3.3±2.5 | –1.42 | –2.33 to –0.51 | –1.03 | 0.01 | –1.84 to –0.22 | –1.13 | 0.01 | | –1.94 to –0.33 |  |
| Lean | Fat mass on electrical impedance as %  of body weight | 15.6±5.3 | 15.0±6.0 | –0.6±3.8 | 15.7±5.3 | | 16.0±6.2 | | ‑0.2±3.8 | –0.78 | –1.55 to –0.01 | Ref | Ref | Ref | Ref | Ref | | Ref |  |
| Overweight | Fat mass on electrical impedance as %  of body weight | 26.0±4.5 | 25.3±5.7 | –0.6±3.4 | 24.7±5.9 | | 26.3±6.4 | | 1.7±3.6 | –2.29 | –3.75 to –0.82 | –1.51 | 0.09 | –3.23 to 0.21 | –1.50 | 0.09 | | –3.22 to 0.21 |  |
| Lean | Energy intake (kcal) ^f^ | 1873±249 | 2058±276 | 185±121 | 1879±241 | | 2089±289 | | 210±120 | -25 | -52 to 2 | Ref | Ref | Ref | Ref | Ref | | Ref |  |
| Overweight | Energy intake (kcal) | 2335±336 | 2535±346 | 200±146 | 2308±317 | | 2661±347 | | 352±197 | -153 | -229 to -76 | -128 | 0.000 | -192 to -63 | -127 | 0.000 | | -193 to -62 |  |
| ^a^  We divided the children in lean or overweight based on the cutoff points of Cole et al. [[2](#_ENREF_2),[3](#_ENREF_3)] Lean children refer to children with a low or healthy BMI (sugar‑free treatment N=179; sugar treatment N=207), overweight children to children with an overweight or obese BMI (sugar‑free treatment N=46; sugar treatment N = 45). Values are mean±SD, unless otherwise indicated  ^b^ We examined the difference in study outcome between lean and overweight children by linear regression using SPSS 20.0. Values are  regression coefficients. The B refers to the differences in changes from baseline in the sugar‑free treatment relative to sugar treatment  ^c^ Model 1 was the crude model, Model 2 was adjusted for age, gender, parental education (lower to high school education, or college/ university education) and parental ethnicity (Dutch, or non–western).  ^d^ Values are regression coefficients (p-value). The B refers to the change from baseline in lean children relative to overweight children, and indicates the interaction term that multiplied treatment (0 or 1) with the initial BMI (0 or 1); P≤0.05 was considered to indicate significance.  ^e^ We calculated z score of body–mass index and height from the Dutch 2009 reference data.[[1](#_ENREF_1)]  ^f^ Since the predictions for energy intake were only possible in children 6 years and over, we could only include 398 children 6-11 years who completed the study. | | | | | | | | | | | | | | | | | | |  |

| **Table C.** Study outcomes of the Double–blind Randomized INtervention study in Kids by baseline BMI. The data refer to the 477 children who remained in treatment for the full 18 months of the study when baseline BMI z score is included as continuous term instead of in categories^a^ | | | |
| --- | --- | --- | --- |
| **Outcome** | **Effect of the treatment per point increase in BMI z score at baseline^b^** | **p-value** | **95% CI** |
| BMI z score^c^ | ‑0.07 | 0.09 | ‑0.14 to 0.01 |
| Body weight (kg) | ‑0.66 | 0.01 | ‑1.12 to ‑0.20 |
| Height (cm) | ‑0.13 | 0.45 | ‑0.47 to 0.21 |
| Sum of four skinfolds (mm) | ‑1.45 | 0.08 | ‑3.09 to 0.18 |
| Waist circumference (cm) | ‑0.62 | 0.02 | ‑1.14 to ‑0.10 |
| Fat mass on electrical impedance (kg | ‑0.30 | 0.07 | ‑0.62 to 0.02 |
| Fat mass on electrical impedance as % of body weight | ‑0.40 | 0.25 | ‑1.09 to 0.28 |
| Energy intake (kcal)^d^ | -34.8 | 0.01 | -61.3 to -8.4 |

^a^ Values are regression coefficients with p-value and 95% confidence intervals. P≤0.05 was considered to indicate statistical significance.

^b^ This model was adjusted for age, gender, parental education (lower to high school education, or college/ university education) and parental ethnicity ((Dutch, or non–western)

^c^ We calculated z score of body–mass index and height from the Dutch 2009 reference data.[[1](#_ENREF_1)]

^d^ Since the predictions for energy intake were only possible in children 6 years and over, we could only include 398 children.

| **Table D.** Responses of participants who finished the trial to the question which type of beverage they thought they had consumed, by baseline BMI.^a^ | | | | | | | |
| --- | --- | --- | --- | --- | --- | --- | --- |
|  | **Response** | | | | |  |  |
|  | ‘Sweetened with artificial sweeteners’ |  | ‘Sweetened with sugar’ |  | ‘I don't know’ or equivalent |  | Subtotals ^b^ |
| **Lower BMI**^c^ |  |  |  |  |  |  |  |
| Sugar‑free | 35 (33%) |  | 19 (18%) |  | 51 (48%) |  |  |
| Sugar | 34 (26%) |  | 27 (21%) |  | 71 (54%) |  |  |
| Total | 69 |  | 46 |  | 122 |  | **237** |
| **Higher BMI** |  |  |  |  |  |  |  |
| Sugar‑free | 46 (39%) |  | 15 (13%) |  | 56 (48%) |  |  |
| Sugar | 33 (28%) |  | 33 (28%) |  | 54 (45%) |  |  |
| Total | 79 |  | 48 |  | 110 |  | **237** |
| ^a^ Values are numbers of children, with percentage of subtotal in parentheses. At the final visit at 18 months we asked available children (*in Dutch*): ‘Alle kinderen hebben dezelfde smaken limonade gedronken: perzik, framboos, mango en citroen. De limonades die door de kinderen werden gedronken waren allemaal zoet. Sommige kinderen hebben altijd limonade gedronken dat zoet was gemaakt met suiker. Andere kinderen hebben altijd limonade gedronken dat zoet was gemaakt met kunstmatige zoetstoffen. Welke limonade heb jij gedronken: limonade met suiker of limonade met kunstmatige zoetstof?’ *English translation*: ‘All children have consumed nonfizzy softdrinks with the same tastes: peach, raspberry, mango and lemon. All the drinks that the children drank were sweet. Some children always drank drinks that had been sweetened with sugar. Other children always drank drinks that had been sweetened with artificial sweeteners. Which drink did you consume: drinks sweetened with sugar or drinks with artificial sweetener?’  ^b^ By mistake we did not administer the questionnaire to 3 children.  ^c^ We divided the children in lower versus higher BMI based on median of the initial BMI z score. A lower BMI refers to children with a BMI z score ≤ ‑0.03 (sugar‑free treatment N=107; sugar treatment N=132), a higher BMI refers to children with a BMI z score > ‑0.03 (sugar‑free treatment N=118; sugar treatment N = 120). | | | | | | | |

| **Table E.** Body weight (mean ± SD) of the 398 children aged 6-11 y for whom the model of Hall et al. [[4](#_ENREF_4)]^a^ et al has been validated. All children completed the Double–blind Randomized INtervention study in Kids. They were classified as having a lower (n=199) or a higher initial BMI (n=199). | | | | | | |
| --- | --- | --- | --- | --- | --- | --- |
|  | **Sugar‑free treatment (n= 185)^b^** | | | **Sugar treatment (n= 213)^b^** | | |
|  | **0 Mo** | **18 Mo** | **Change** | **0 Mo** | **18 Mo** | **Change** |
| *Body weight (kg)* |  |  |  |  |  |  |
| All | 31.7 ± 8.0 | 38.3 ± 9.7 | 6.7 ± 2.8 | 31.4 ± 7.8 | 39.0 ± 10.3 | 7.6 ± 3.5 |
| Lower BMI | 27.2 ± 5.1 | 32.8 ± 6.3 | 5.6 ± 2.3 | 27.8 ± 5.3 | 34.1 ± 7.5 | 6.3 ± 2.8 |
| Higher BMI | 35.7 ± 8.0 | 43.4 ± 9.6 | 7.6 ± 2.9 | 35.3 ± 8.2 | 44.2 ± 10.5 | 8.9 ± 3.6 |
| ^a^ The model [[4](#_ENREF_4)] has been validated only for children aged 6 years and over. Therefore the calculation of caloric compensation was limited to the 398 children aged 6-11 who completed the study. As a result numbers differ from those in table 2.  ^b^ Within the sugar‑free treatment 88 children had a lower and 97 children a higher initial BMI; within the sugar treatment 111 children had a lower and 102 children a higher initial BMI. | | | | | | |

**Table F.** The full dataset of predicted energy intakes of the 398 children aged 6-11 y for whom the model of Hall et al.[1] has been validated.

| **Unique identification number** | **Baseline BMI (Low =0; high =1)** | **Treatment (sugar-free = 0; sugar = 1)** | **Gender (1= boy; 2 = girl)** | **Age (in years)** | **Body weight (baseline)** | **Body weight (18 months)** | **Completed 18 months study period (1=yes; 0=no)** | **Intake (kCal) Initial** | **Intake (kCal) 18months** | **Delta Energy intake (kCal)** |
| --- | --- | --- | --- | --- | --- | --- | --- | --- | --- | --- |
| 1096 | 0 | 0 | 2 | 7,21 | 20,7 | 25,1 | 1 | 1435,37 | 1605,99 | 170,62 |
| 1108 | 0 | 0 | 2 | 9,33 | 37,35 | 40 | 1 | 2120,11 | 2032,70 | -87,42 |
| 1111 | 0 | 0 | 1 | 7,31 | 21,4 | 25 | 1 | 1604,33 | 1748,25 | 143,91 |
| 1113 | 0 | 0 | 1 | 6,52 | 23,1 | 28,8 | 1 | 1722,92 | 1975,72 | 252,81 |
| 1132 | 0 | 0 | 1 | 9,38 | 30,2 | 33,15 | 1 | 1972,08 | 1964,86 | -7,22 |
| 1133 | 0 | 0 | 1 | 6,72 | 21,9 | 25,6 | 1 | 1651,90 | 1801,01 | 149,11 |
| 1135 | 0 | 0 | 1 | 9,4 | 31,6 | 37,9 | 1 | 2035,80 | 2198,60 | 162,80 |
| 1213 | 0 | 0 | 2 | 6,68 | 22,8 | 29 | 1 | 1561,49 | 1820,65 | 259,15 |
| 1234 | 0 | 0 | 2 | 7,15 | 24,65 | 29,55 | 1 | 1639,57 | 1809,10 | 169,53 |
| 1252 | 0 | 0 | 2 | 8,56 | 30,3 | 46,9 | 1 | 1859,81 | 2522,21 | 662,41 |
| 1259 | 0 | 0 | 2 | 7,85 | 25,85 | 35,7 | 1 | 1673,55 | 2061,10 | 387,54 |
| 1310 | 0 | 0 | 1 | 9,32 | 29,8 | 34,85 | 1 | 1957,01 | 2062,66 | 105,65 |
| 1342 | 0 | 0 | 2 | 10,82 | 26,5 | 29,9 | 1 | 1506,95 | 1556,49 | 49,53 |
| 1347 | 0 | 0 | 1 | 9,97 | 36,5 | 38,3 | 1 | 2220,47 | 2104,05 | -116,42 |
| 1474 | 0 | 0 | 1 | 6,33 | 19 | 24,8 | 1 | 1500,20 | 1788,66 | 288,46 |
| 1482 | 0 | 0 | 1 | 6,45 | 17,9 | 23,65 | 1 | 1435,68 | 1728,06 | 292,38 |
| 1520 | 0 | 0 | 1 | 7,79 | 22,4 | 31,6 | 1 | 1643,57 | 2075,86 | 432,28 |
| 1564 | 0 | 0 | 1 | 7,39 | 21,35 | 26 | 1 | 1598,50 | 1799,12 | 200,62 |
| 1618 | 0 | 0 | 1 | 6,94 | 25,4 | 30,2 | 1 | 1834,84 | 2020,47 | 185,64 |
| 1651 | 0 | 0 | 1 | 8,08 | 32,45 | 39,4 | 1 | 2152,87 | 2394,61 | 241,74 |
| 1726 | 0 | 0 | 1 | 9,02 | 26,6 | 31,8 | 1 | 1815,69 | 1961,24 | 145,55 |
| 1785 | 0 | 0 | 1 | 10,69 | 36,05 | 44,5 | 1 | 2132,49 | 2335,17 | 202,67 |
| 1795 | 0 | 0 | 1 | 10,95 | 31,7 | 38,2 | 1 | 1914,84 | 2048,04 | 133,20 |
| 1805 | 0 | 0 | 1 | 11,52 | 29,95 | 36,95 | 1 | 1780,27 | 1948,31 | 168,04 |
| 1819 | 0 | 0 | 2 | 10,23 | 29,6 | 34,85 | 1 | 1687,44 | 1801,62 | 114,18 |
| 1830 | 0 | 0 | 1 | 10,76 | 33,3 | 41,2 | 1 | 2002,41 | 2194,70 | 192,29 |
| 1833 | 0 | 0 | 2 | 11,68 | 39 | 48,05 | 1 | 1971,29 | 2220,90 | 249,60 |
| 2112 | 0 | 0 | 2 | 6,63 | 22 | 26,45 | 1 | 1523,27 | 1695,15 | 171,88 |
| 2119 | 0 | 0 | 2 | 6,51 | 20,45 | 26,15 | 1 | 1446,55 | 1691,96 | 245,41 |
| 2130 | 0 | 0 | 2 | 7,47 | 18,4 | 22 | 1 | 1315,67 | 1440,65 | 124,98 |
| 2136 | 0 | 0 | 1 | 8,83 | 25,1 | 31,6 | 1 | 1751,70 | 1971,02 | 219,32 |
| 2160 | 0 | 0 | 1 | 9,17 | 25,2 | 28,6 | 1 | 1739,50 | 1782,11 | 42,62 |
| 2162 | 0 | 0 | 1 | 8,61 | 28,6 | 32,4 | 1 | 1934,20 | 1999,92 | 65,72 |
| 2178 | 0 | 0 | 1 | 9,54 | 26,65 | 29,6 | 1 | 1787,36 | 1791,80 | 4,44 |
| 2559 | 0 | 0 | 2 | 6,53 | 20,9 | 26,15 | 1 | 1466,79 | 1690,37 | 223,58 |
| 2606 | 0 | 0 | 2 | 7,29 | 23,6 | 27,3 | 1 | 1580,67 | 1693,27 | 112,60 |
| 2646 | 0 | 0 | 2 | 8,22 | 24,3 | 27,7 | 1 | 1588,28 | 1625,18 | 36,91 |
| 2657 | 0 | 0 | 1 | 8,32 | 28,25 | 30,75 | 1 | 1926,41 | 1941,60 | 15,18 |
| 2663 | 0 | 0 | 1 | 9,48 | 25,05 | 28,5 | 1 | 1712,40 | 1754,88 | 42,49 |
| 2726 | 0 | 0 | 2 | 11,21 | 33,3 | 36,7 | 1 | 1774,40 | 1790,47 | 16,07 |
| 2727 | 0 | 0 | 2 | 10,95 | 31,5 | 38 | 1 | 1717,16 | 1888,16 | 171,00 |
| 2736 | 0 | 0 | 2 | 10,64 | 37,85 | 47,5 | 1 | 2005,03 | 2288,08 | 283,05 |
| 3050 | 0 | 0 | 1 | 10,46 | 31,9 | 40,1 | 1 | 1963,86 | 2186,35 | 222,50 |
| 3107 | 0 | 0 | 1 | 6,19 | 18,65 | 22,3 | 1 | 1482,97 | 1655,48 | 172,51 |
| 3134 | 0 | 0 | 2 | 6,96 | 21,6 | 25,85 | 1 | 1494,96 | 1651,20 | 156,24 |
| 3170 | 0 | 0 | 2 | 7,72 | 26,15 | 34,6 | 1 | 1689,39 | 2017,44 | 328,06 |
| 3171 | 0 | 0 | 2 | 7,61 | 23,15 | 29,35 | 1 | 1554,30 | 1778,95 | 224,65 |
| 3178 | 0 | 0 | 1 | 7,52 | 25,5 | 33,55 | 1 | 1815,47 | 2178,15 | 362,69 |
| 3184 | 0 | 0 | 2 | 7,95 | 26,2 | 31,4 | 1 | 1687,81 | 1824,00 | 136,19 |
| 3189 | 0 | 0 | 1 | 7,04 | 24,95 | 28 | 1 | 1801,46 | 1895,54 | 94,07 |
| 3195 | 0 | 0 | 2 | 7,62 | 27,9 | 34,9 | 1 | 1782,63 | 2025,43 | 242,80 |
| 3202 | 0 | 0 | 2 | 6,96 | 21,5 | 25,5 | 1 | 1486,48 | 1633,90 | 147,41 |
| 3225 | 0 | 0 | 1 | 7,97 | 22,3 | 27,65 | 1 | 1637,81 | 1845,91 | 208,10 |
| 3246 | 0 | 0 | 1 | 8,22 | 25,9 | 31,1 | 1 | 1815,45 | 1987,95 | 172,50 |
| 3249 | 0 | 0 | 1 | 8,74 | 27,05 | 32,1 | 1 | 1856,67 | 1989,47 | 132,80 |
| 3256 | 0 | 0 | 1 | 8,45 | 27,7 | 31,3 | 1 | 1896,50 | 1960,39 | 63,89 |
| 3259 | 0 | 0 | 1 | 8,5 | 27,4 | 33,05 | 1 | 1880,32 | 2059,70 | 179,38 |
| 3272 | 0 | 0 | 1 | 9,36 | 31,8 | 37,4 | 1 | 2047,40 | 2174,58 | 127,18 |
| 3275 | 0 | 0 | 1 | 9,63 | 27,3 | 33,8 | 1 | 1812,75 | 1999,17 | 186,42 |
| 3281 | 0 | 0 | 2 | 9,75 | 32,1 | 41,3 | 1 | 1844,11 | 2129,53 | 285,41 |
| 3285 | 0 | 0 | 2 | 9,56 | 29,6 | 37,4 | 1 | 1748,00 | 1973,61 | 225,61 |
| 3286 | 0 | 0 | 1 | 9,51 | 28,55 | 33,6 | 1 | 1880,23 | 1992,27 | 112,04 |
| 3307 | 0 | 0 | 2 | 9,46 | 27,95 | 32,25 | 1 | 1686,97 | 1740,05 | 53,08 |
| 3327 | 0 | 0 | 1 | 10,73 | 34,5 | 45,1 | 1 | 2059,90 | 2377,65 | 317,75 |
| 3329 | 0 | 0 | 1 | 10,46 | 35,1 | 42,6 | 1 | 2111,56 | 2280,07 | 168,51 |
| 4225 | 0 | 0 | 1 | 6,05 | 20,05 | 25,45 | 1 | 1567,51 | 1825,48 | 257,97 |
| 5317 | 0 | 0 | 1 | 6,09 | 24,15 | 29,5 | 1 | 1807,47 | 2017,97 | 210,50 |
| 5374 | 0 | 0 | 1 | 6,96 | 23,45 | 27,9 | 1 | 1727,91 | 1904,48 | 176,57 |
| 5418 | 0 | 0 | 2 | 8,1 | 29 | 36,15 | 1 | 1818,88 | 2031,00 | 212,12 |
| 5428 | 0 | 0 | 1 | 7,88 | 23,45 | 26,7 | 1 | 1698,89 | 1790,42 | 91,53 |
| 5430 | 0 | 0 | 2 | 7,81 | 22,4 | 26,6 | 1 | 1505,85 | 1618,98 | 113,13 |
| 5434 | 0 | 0 | 2 | 7,69 | 28 | 33,55 | 1 | 1785,00 | 1944,21 | 159,21 |
| 5459 | 0 | 0 | 1 | 8,75 | 27,2 | 35,5 | 1 | 1857,76 | 2170,88 | 313,13 |
| 5475 | 0 | 0 | 1 | 11,17 | 39,1 | 46,95 | 1 | 2211,95 | 2364,74 | 152,79 |
| 5479 | 0 | 0 | 1 | 11,07 | 39 | 46,2 | 1 | 2223,53 | 2341,84 | 118,31 |
| 5532 | 0 | 0 | 1 | 8,9 | 28,6 | 33,6 | 1 | 1923,46 | 2043,73 | 120,27 |
| 5567 | 0 | 0 | 2 | 7,57 | 20,75 | 26,2 | 1 | 1431,69 | 1637,48 | 205,80 |
| 5594 | 0 | 0 | 1 | 10,01 | 35,3 | 42,7 | 1 | 2161,48 | 2335,53 | 174,05 |
| 5598 | 0 | 0 | 2 | 8,86 | 32,6 | 35,15 | 1 | 1948,63 | 1873,45 | -75,17 |
| 5611 | 0 | 0 | 2 | 9,44 | 22,25 | 25,6 | 1 | 1423,14 | 1456,93 | 33,79 |
| 5613 | 0 | 0 | 1 | 11,62 | 29,95 | 34,85 | 1 | 1771,12 | 1839,09 | 67,97 |
| 5621 | 0 | 0 | 1 | 8,9 | 27,95 | 32,7 | 1 | 1891,58 | 2001,12 | 109,53 |
| 5630 | 0 | 0 | 1 | 7,57 | 27,5 | 33,05 | 1 | 1921,26 | 2129,88 | 208,62 |
| 5656 | 0 | 0 | 1 | 7,14 | 23,9 | 27,2 | 1 | 1745,79 | 1856,85 | 111,06 |
| 5940 | 0 | 0 | 1 | 8,01 | 25,8 | 32,8 | 1 | 1817,41 | 2096,40 | 278,99 |
| 5941 | 0 | 0 | 2 | 6,96 | 24,8 | 31,5 | 1 | 1655,24 | 1922,66 | 267,42 |
| 5985 | 0 | 0 | 1 | 8,67 | 28,7 | 34,45 | 1 | 1936,92 | 2112,73 | 175,81 |
| 6061 | 0 | 0 | 1 | 8,72 | 25,05 | 29,75 | 1 | 1748,72 | 1882,51 | 133,79 |
| 1095 | 0 | 1 | 1 | 8,7 | 26,8 | 34,75 | 1 | 1842,57 | 2133,49 | 290,92 |
| 1116 | 0 | 1 | 2 | 8,82 | 25,2 | 29,1 | 1 | 1608,08 | 1636,51 | 28,43 |
| 1118 | 0 | 1 | 1 | 9,16 | 31 | 37,3 | 1 | 2022,90 | 2195,87 | 172,97 |
| 1122 | 0 | 1 | 2 | 6,02 | 20,45 | 23,8 | 1 | 1467,99 | 1582,90 | 114,91 |
| 1123 | 0 | 1 | 1 | 9,92 | 33,95 | 42,2 | 1 | 2103,39 | 2334,03 | 230,64 |
| 1136 | 0 | 1 | 1 | 9,32 | 33,6 | 38,8 | 1 | 2140,75 | 2232,01 | 91,26 |
| 1184 | 0 | 1 | 1 | 6,24 | 21,6 | 26,15 | 1 | 1651,25 | 1847,83 | 196,58 |
| 1229 | 0 | 1 | 1 | 6,95 | 22,7 | 27,4 | 1 | 1688,52 | 1886,10 | 197,58 |
| 1232 | 0 | 1 | 2 | 6,59 | 18,1 | 23,3 | 1 | 1319,70 | 1559,41 | 239,71 |
| 1262 | 0 | 1 | 2 | 8,76 | 25,9 | 31,5 | 1 | 1640,00 | 1761,97 | 121,97 |
| 1266 | 0 | 1 | 1 | 8,02 | 25,45 | 30,9 | 1 | 1799,44 | 1996,69 | 197,25 |
| 1337 | 0 | 1 | 1 | 9,84 | 32,1 | 44,05 | 1 | 2026,77 | 2459,09 | 432,32 |
| 1353 | 0 | 1 | 2 | 11,62 | 39,6 | 50,7 | 1 | 2000,25 | 2341,32 | 341,07 |
| 1355 | 0 | 1 | 2 | 10,7 | 36,15 | 47,2 | 1 | 1930,41 | 2292,04 | 361,63 |
| 1372 | 0 | 1 | 2 | 10,65 | 39,2 | 50 | 1 | 2057,74 | 2392,99 | 335,25 |
| 1373 | 0 | 1 | 1 | 11,61 | 37,35 | 50 | 1 | 2091,11 | 2488,14 | 397,03 |
| 1550 | 0 | 1 | 1 | 7,58 | 25,8 | 33 | 1 | 1828,58 | 2138,65 | 310,06 |
| 1579 | 0 | 1 | 1 | 7,17 | 25 | 30,9 | 1 | 1800,25 | 2053,18 | 252,93 |
| 1604 | 0 | 1 | 2 | 7,44 | 26,8 | 33,6 | 1 | 1735,31 | 1983,85 | 248,55 |
| 1607 | 0 | 1 | 2 | 7,88 | 23,3 | 28,75 | 1 | 1550,10 | 1720,16 | 170,06 |
| 1624 | 0 | 1 | 1 | 8,9 | 24,15 | 29 | 1 | 1700,78 | 1835,06 | 134,28 |
| 1685 | 0 | 1 | 1 | 8,17 | 25,55 | 34,85 | 1 | 1799,47 | 2198,73 | 399,26 |
| 1688 | 0 | 1 | 1 | 8,36 | 22,4 | 28,2 | 1 | 1630,76 | 1844,63 | 213,87 |
| 1692 | 0 | 1 | 1 | 8,92 | 29,8 | 35,2 | 1 | 1979,55 | 2117,16 | 137,61 |
| 1713 | 0 | 1 | 2 | 9,97 | 25,75 | 34,8 | 1 | 1536,48 | 1856,57 | 320,09 |
| 1715 | 0 | 1 | 2 | 9,73 | 27,05 | 31,45 | 1 | 1619,89 | 1693,25 | 73,36 |
| 1781 | 0 | 1 | 2 | 10,91 | 36,5 | 46,25 | 1 | 1928,89 | 2222,07 | 293,19 |
| 1784 | 0 | 1 | 1 | 10,28 | 35,4 | 41,1 | 1 | 2139,23 | 2223,24 | 84,02 |
| 1794 | 0 | 1 | 2 | 11,2 | 32,75 | 39,55 | 1 | 1752,24 | 1931,11 | 178,87 |
| 1820 | 0 | 1 | 2 | 10,75 | 35,65 | 47,1 | 1 | 1904,92 | 2289,22 | 384,30 |
| 1822 | 0 | 1 | 1 | 10,56 | 31,25 | 41,15 | 1 | 1928,02 | 2236,04 | 308,02 |
| 2083 | 0 | 1 | 1 | 6,16 | 23,2 | 28,35 | 1 | 1742,21 | 1962,88 | 220,68 |
| 2105 | 0 | 1 | 1 | 6,88 | 19,9 | 23,8 | 1 | 1538,11 | 1706,40 | 168,29 |
| 2132 | 0 | 1 | 1 | 7,93 | 26,75 | 31,05 | 1 | 1865,92 | 2000,07 | 134,15 |
| 2143 | 0 | 1 | 2 | 8,38 | 25,65 | 33 | 1 | 1648,47 | 1872,02 | 223,55 |
| 2147 | 0 | 1 | 2 | 8,85 | 23,75 | 29,4 | 1 | 1537,95 | 1667,04 | 129,09 |
| 2179 | 0 | 1 | 2 | 9,72 | 32,1 | 37,55 | 1 | 1847,45 | 1945,79 | 98,34 |
| 2195 | 0 | 1 | 1 | 10,58 | 35,3 | 45,9 | 1 | 2109,83 | 2426,91 | 317,08 |
| 2203 | 0 | 1 | 2 | 10,83 | 35,25 | 47,6 | 1 | 1881,25 | 2311,19 | 429,94 |
| 2541 | 0 | 1 | 1 | 6,05 | 23,2 | 29,5 | 1 | 1754,19 | 2027,64 | 273,45 |
| 2558 | 0 | 1 | 1 | 6,91 | 23,55 | 26,1 | 1 | 1734,49 | 1805,65 | 71,16 |
| 2565 | 0 | 1 | 1 | 6,73 | 22,15 | 26,1 | 1 | 1665,36 | 1826,59 | 161,24 |
| 2576 | 0 | 1 | 2 | 6,5 | 24,2 | 29,5 | 1 | 1646,46 | 1841,74 | 195,29 |
| 2579 | 0 | 1 | 1 | 7,12 | 22,25 | 26,75 | 1 | 1658,72 | 1846,50 | 187,78 |
| 2580 | 0 | 1 | 1 | 6,6 | 27,25 | 34,1 | 1 | 1951,33 | 2233,75 | 282,41 |
| 2587 | 0 | 1 | 1 | 6,58 | 22,7 | 25,8 | 1 | 1699,71 | 1809,99 | 110,28 |
| 2611 | 0 | 1 | 1 | 7,5 | 22,6 | 26,9 | 1 | 1665,30 | 1835,43 | 170,13 |
| 2624 | 0 | 1 | 2 | 7,44 | 20,7 | 26 | 1 | 1427,27 | 1642,41 | 215,14 |
| 2684 | 0 | 1 | 2 | 9,91 | 29,1 | 33,45 | 1 | 1694,14 | 1761,13 | 67,00 |
| 2701 | 0 | 1 | 1 | 11,06 | 34,6 | 43,55 | 1 | 2032,03 | 2262,43 | 230,40 |
| 2705 | 0 | 1 | 1 | 10,26 | 32,5 | 36,9 | 1 | 2010,70 | 2042,36 | 31,66 |
| 3024 | 0 | 1 | 1 | 10,7 | 34,3 | 41,2 | 1 | 2057,69 | 2191,16 | 133,47 |
| 3047 | 0 | 1 | 2 | 10,96 | 32,7 | 43,1 | 1 | 1767,48 | 2116,17 | 348,69 |
| 3048 | 0 | 1 | 1 | 10,4 | 29 | 34,75 | 1 | 1836,14 | 1955,37 | 119,23 |
| 3113 | 0 | 1 | 1 | 6,09 | 21,1 | 25,9 | 1 | 1628,31 | 1840,20 | 211,89 |
| 3144 | 0 | 1 | 1 | 7,27 | 18,6 | 22 | 1 | 1458,65 | 1600,13 | 141,48 |
| 3153 | 0 | 1 | 1 | 6,12 | 24,55 | 32,4 | 1 | 1828,81 | 2175,48 | 346,67 |
| 3180 | 0 | 1 | 1 | 7,99 | 21,35 | 24,7 | 1 | 1587,58 | 1689,23 | 101,65 |
| 3181 | 0 | 1 | 1 | 7,25 | 27 | 31,2 | 1 | 1907,57 | 2046,73 | 139,16 |
| 3183 | 0 | 1 | 2 | 9,18 | 20,2 | 25,1 | 1 | 1345,18 | 1458,63 | 113,45 |
| 3186 | 0 | 1 | 2 | 7,89 | 21,9 | 24,5 | 1 | 1482,04 | 1509,91 | 27,87 |
| 3196 | 0 | 1 | 1 | 7,78 | 26,05 | 32,25 | 1 | 1835,78 | 2083,59 | 247,81 |
| 3237 | 0 | 1 | 1 | 9,5 | 26,9 | 32,3 | 1 | 1802,51 | 1939,29 | 136,78 |
| 3254 | 0 | 1 | 1 | 8,89 | 30,15 | 35,2 | 1 | 1996,74 | 2116,36 | 119,62 |
| 3268 | 0 | 1 | 1 | 9,37 | 27,6 | 35,05 | 1 | 1842,00 | 2085,67 | 243,68 |
| 3288 | 0 | 1 | 1 | 9,77 | 32,4 | 41,7 | 1 | 2046,05 | 2345,00 | 298,95 |
| 3291 | 0 | 1 | 1 | 10,78 | 26,85 | 33,1 | 1 | 1705,04 | 1854,56 | 149,52 |
| 3299 | 0 | 1 | 2 | 9,38 | 31,9 | 37,1 | 1 | 1873,38 | 1947,28 | 73,90 |
| 3310 | 0 | 1 | 2 | 10,78 | 32,95 | 51,1 | 1 | 1789,79 | 2505,92 | 716,13 |
| 3312 | 0 | 1 | 1 | 10,55 | 34,6 | 39,9 | 1 | 2081,64 | 2140,65 | 59,01 |
| 3321 | 0 | 1 | 2 | 10,15 | 32,85 | 41,5 | 1 | 1834,89 | 2096,36 | 261,47 |
| 3322 | 0 | 1 | 2 | 10,76 | 40,5 | 56,3 | 1 | 2104,93 | 2663,52 | 558,59 |
| 5326 | 0 | 1 | 1 | 6,52 | 24,15 | 29,9 | 1 | 1783,76 | 2029,52 | 245,77 |
| 5350 | 0 | 1 | 2 | 6,83 | 18,25 | 21,8 | 1 | 1321,98 | 1468,75 | 146,76 |
| 5398 | 0 | 1 | 1 | 8,88 | 26,95 | 31,9 | 1 | 1841,83 | 1969,16 | 127,33 |
| 5426 | 0 | 1 | 2 | 7,9 | 22,4 | 27,9 | 1 | 1502,90 | 1682,27 | 179,37 |
| 5440 | 0 | 1 | 1 | 7,17 | 25,3 | 29,05 | 1 | 1820,57 | 1946,22 | 125,65 |
| 5464 | 0 | 1 | 1 | 8,5 | 27,8 | 34,9 | 1 | 1899,71 | 2152,44 | 252,74 |
| 5466 | 0 | 1 | 1 | 8,49 | 25,8 | 29,4 | 1 | 1800,04 | 1870,88 | 70,83 |
| 5467 | 0 | 1 | 1 | 8,42 | 24,85 | 29,6 | 1 | 1752,65 | 1898,45 | 145,80 |
| 5493 | 0 | 1 | 1 | 10,24 | 33,8 | 40,2 | 1 | 2072,35 | 2199,09 | 126,74 |
| 5521 | 0 | 1 | 1 | 9,84 | 32,6 | 37,5 | 1 | 2049,73 | 2118,81 | 69,08 |
| 5545 | 0 | 1 | 2 | 10,96 | 34,7 | 41,35 | 1 | 1851,29 | 2009,71 | 158,42 |
| 5580 | 0 | 1 | 1 | 10,04 | 28,2 | 31,75 | 1 | 1824,88 | 1842,86 | 17,98 |
| 5581 | 0 | 1 | 1 | 9,13 | 28,4 | 32,75 | 1 | 1901,00 | 1980,08 | 79,08 |
| 5587 | 0 | 1 | 1 | 11,05 | 32,3 | 38,05 | 1 | 1932,32 | 2021,31 | 88,99 |
| 5588 | 0 | 1 | 2 | 10,96 | 30,3 | 40,5 | 1 | 1665,40 | 2018,39 | 352,99 |
| 5593 | 0 | 1 | 1 | 9,73 | 32,3 | 39,05 | 1 | 2044,66 | 2214,26 | 169,61 |
| 5595 | 0 | 1 | 1 | 9,57 | 31,4 | 36,5 | 1 | 2015,05 | 2109,65 | 94,60 |
| 5597 | 0 | 1 | 2 | 10,99 | 33,45 | 43,8 | 1 | 1797,14 | 2138,75 | 341,62 |
| 5603 | 0 | 1 | 1 | 8,19 | 28,35 | 34,35 | 1 | 1941,54 | 2146,20 | 204,67 |
| 5607 | 0 | 1 | 1 | 8,58 | 31,3 | 37,85 | 1 | 2072,66 | 2273,39 | 200,72 |
| 5615 | 0 | 1 | 1 | 7,93 | 25,7 | 28,9 | 1 | 1814,64 | 1887,46 | 72,82 |
| 5616 | 0 | 1 | 2 | 8,11 | 27,8 | 31,5 | 1 | 1761,46 | 1801,08 | 39,62 |
| 5625 | 0 | 1 | 1 | 10,6 | 32 | 38,5 | 1 | 1955,26 | 2093,96 | 138,69 |
| 5626 | 0 | 1 | 2 | 7,07 | 19,1 | 23,4 | 1 | 1360,28 | 1536,10 | 175,82 |
| 5628 | 0 | 1 | 1 | 6,74 | 23,25 | 26,7 | 1 | 1723,83 | 1850,65 | 126,82 |
| 5632 | 0 | 1 | 1 | 7 | 22,4 | 27,3 | 1 | 1672,75 | 1880,05 | 207,30 |
| 5637 | 0 | 1 | 1 | 7,07 | 21,75 | 26,75 | 1 | 1632,60 | 1850,61 | 218,00 |
| 5639 | 0 | 1 | 1 | 6,74 | 22,7 | 27,5 | 1 | 1694,75 | 1899,53 | 204,78 |
| 5642 | 0 | 1 | 1 | 6,38 | 23,65 | 27,1 | 1 | 1756,62 | 1880,38 | 123,76 |
| 5650 | 0 | 1 | 1 | 7,28 | 23,1 | 27,5 | 1 | 1694,78 | 1875,76 | 180,98 |
| 5651 | 0 | 1 | 1 | 7,15 | 25,85 | 31,7 | 1 | 1850,58 | 2091,22 | 240,64 |
| 5654 | 0 | 1 | 2 | 7,41 | 27,3 | 33,4 | 1 | 1761,07 | 1969,67 | 208,60 |
| 5945 | 0 | 1 | 2 | 7,58 | 24,25 | 29,8 | 1 | 1604,28 | 1795,66 | 191,38 |
| 5946 | 0 | 1 | 1 | 7,92 | 28,75 | 35,55 | 1 | 1972,58 | 2229,85 | 257,27 |
| 5956 | 0 | 1 | 2 | 10,27 | 40,5 | 52,8 | 1 | 2150,57 | 2544,49 | 393,92 |
| 5957 | 0 | 1 | 1 | 8,32 | 26,1 | 31,45 | 1 | 1822,07 | 1997,06 | 174,99 |
| 5965 | 0 | 1 | 1 | 7,64 | 24,5 | 28 | 1 | 1762,06 | 1870,25 | 108,19 |
| 6066 | 0 | 1 | 2 | 7,66 | 29,85 | 36,8 | 1 | 1875,43 | 2100,34 | 224,91 |
| 6067 | 0 | 1 | 1 | 9,44 | 29,5 | 33,8 | 1 | 1933,80 | 1998,74 | 64,94 |
| 1098 | 1 | 0 | 1 | 8,85 | 36,8 | 42,8 | 1 | 2326,01 | 2454,52 | 128,51 |
| 1199 | 1 | 0 | 2 | 6,2 | 22,8 | 30,6 | 1 | 1588,98 | 1916,64 | 327,66 |
| 1238 | 1 | 0 | 2 | 6,96 | 27,35 | 31,1 | 1 | 1784,64 | 1878,12 | 93,47 |
| 1289 | 1 | 0 | 2 | 7,91 | 30,4 | 38,6 | 1 | 1892,57 | 2164,04 | 271,48 |
| 1291 | 1 | 0 | 2 | 8,2 | 33,45 | 40,8 | 1 | 2023,28 | 2212,40 | 189,12 |
| 1327 | 1 | 0 | 1 | 9,66 | 46,7 | 58,65 | 1 | 2711,31 | 3052,42 | 341,11 |
| 1336 | 1 | 0 | 1 | 9,24 | 38,5 | 51,75 | 1 | 2378,77 | 2863,57 | 484,79 |
| 1343 | 1 | 0 | 2 | 10,82 | 39 | 49,3 | 1 | 2037,26 | 2343,37 | 306,11 |
| 1349 | 1 | 0 | 2 | 10,37 | 42,85 | 47 | 1 | 2237,19 | 2224,41 | -12,78 |
| 1551 | 1 | 0 | 2 | 7,61 | 29,5 | 35,4 | 1 | 1860,47 | 2033,50 | 173,03 |
| 1584 | 1 | 0 | 1 | 8,44 | 35,5 | 42,6 | 1 | 2285,56 | 2497,92 | 212,37 |
| 1594 | 1 | 0 | 1 | 7,04 | 29,35 | 39,65 | 1 | 2041,28 | 2503,30 | 462,02 |
| 1609 | 1 | 0 | 1 | 7,44 | 23,85 | 27,3 | 1 | 1734,28 | 1849,31 | 115,02 |
| 1629 | 1 | 0 | 2 | 9,11 | 33,8 | 35,65 | 1 | 1983,78 | 1869,03 | -114,74 |
| 1677 | 1 | 0 | 2 | 8,02 | 37,35 | 47 | 1 | 2211,94 | 2507,18 | 295,24 |
| 1712 | 1 | 0 | 1 | 9,82 | 35,95 | 46,4 | 1 | 2209,39 | 2541,34 | 331,95 |
| 1717 | 1 | 0 | 1 | 9,58 | 32,7 | 39,65 | 1 | 2073,52 | 2259,27 | 185,75 |
| 1720 | 1 | 0 | 1 | 9,89 | 38,65 | 49,5 | 1 | 2326,84 | 2658,72 | 331,89 |
| 1723 | 1 | 0 | 1 | 9,36 | 35,5 | 47,6 | 1 | 2227,99 | 2667,21 | 439,22 |
| 1797 | 1 | 0 | 1 | 10,75 | 37,7 | 43,85 | 1 | 2201,02 | 2278,18 | 77,16 |
| 1826 | 1 | 0 | 2 | 10,74 | 55,35 | 67,6 | 1 | 2689,71 | 2981,44 | 291,72 |
| 2102 | 1 | 0 | 2 | 7,01 | 26,7 | 34,2 | 1 | 1749,26 | 2044,39 | 295,13 |
| 2134 | 1 | 0 | 2 | 7,13 | 25,9 | 29,1 | 1 | 1703,35 | 1777,25 | 73,90 |
| 2135 | 1 | 0 | 1 | 8,81 | 31,2 | 35,05 | 1 | 2056,77 | 2100,45 | 43,68 |
| 2155 | 1 | 0 | 1 | 8,75 | 29,95 | 35 | 1 | 1997,89 | 2118,32 | 120,43 |
| 2170 | 1 | 0 | 2 | 9,61 | 36,5 | 39,5 | 1 | 2052,96 | 1999,64 | -53,32 |
| 2173 | 1 | 0 | 1 | 10,31 | 47,8 | 53,8 | 1 | 2686,57 | 2695,32 | 8,75 |
| 2180 | 1 | 0 | 1 | 9,59 | 39,5 | 47,5 | 1 | 2394,67 | 2587,46 | 192,79 |
| 2185 | 1 | 0 | 2 | 9,39 | 30 | 38,69 | 1 | 1785,66 | 2042,77 | 257,12 |
| 2187 | 1 | 0 | 1 | 10,44 | 50,4 | 56,2 | 1 | 2780,78 | 2757,52 | -23,26 |
| 2199 | 1 | 0 | 1 | 11,19 | 55,75 | 64,3 | 1 | 2891,35 | 2961,38 | 70,04 |
| 2209 | 1 | 0 | 1 | 11,06 | 50,15 | 56,8 | 1 | 2689,17 | 2703,06 | 13,88 |
| 2568 | 1 | 0 | 2 | 6,06 | 21,8 | 26,1 | 1 | 1539,05 | 1693,37 | 154,32 |
| 2571 | 1 | 0 | 1 | 6,28 | 24,7 | 32,4 | 1 | 1827,18 | 2169,26 | 342,08 |
| 2626 | 1 | 0 | 2 | 7,45 | 28,4 | 35,2 | 1 | 1813,49 | 2050,71 | 237,22 |
| 2645 | 1 | 0 | 1 | 8,34 | 43,1 | 49,65 | 1 | 2659,35 | 2792,17 | 132,82 |
| 2671 | 1 | 0 | 2 | 9,67 | 39,2 | 49,5 | 1 | 2162,72 | 2453,20 | 290,48 |
| 2694 | 1 | 0 | 1 | 8,98 | 42,5 | 50,25 | 1 | 2585,11 | 2767,09 | 181,98 |
| 2708 | 1 | 0 | 2 | 9,98 | 40,1 | 48,3 | 1 | 2164,07 | 2357,87 | 193,79 |
| 2715 | 1 | 0 | 2 | 10,62 | 45,8 | 54,9 | 1 | 2331,14 | 2538,50 | 207,36 |
| 2730 | 1 | 0 | 1 | 10,16 | 36,65 | 42,85 | 1 | 2210,57 | 2310,65 | 100,08 |
| 3026 | 1 | 0 | 1 | 9,05 | 39,2 | 50,5 | 1 | 2426,58 | 2812,72 | 386,15 |
| 3042 | 1 | 0 | 2 | 10,56 | 40,3 | 53,9 | 1 | 2114,31 | 2571,72 | 457,41 |
| 3052 | 1 | 0 | 1 | 10,49 | 39,65 | 51,05 | 1 | 2313,50 | 2642,64 | 329,14 |
| 3111 | 1 | 0 | 2 | 6,21 | 28,2 | 35,4 | 1 | 1885,85 | 2118,92 | 233,07 |
| 3139 | 1 | 0 | 2 | 6,61 | 26,25 | 34,85 | 1 | 1747,64 | 2100,26 | 352,62 |
| 3190 | 1 | 0 | 2 | 7,56 | 25,95 | 31,4 | 1 | 1689,31 | 1864,65 | 175,34 |
| 3192 | 1 | 0 | 1 | 7,68 | 33,4 | 40,1 | 1 | 2218,69 | 2451,26 | 232,57 |
| 3198 | 1 | 0 | 1 | 8,45 | 27,1 | 31,15 | 1 | 1867,33 | 1959,05 | 91,72 |
| 3207 | 1 | 0 | 2 | 7,08 | 28,3 | 36,7 | 1 | 1826,01 | 2155,86 | 329,85 |
| 3226 | 1 | 0 | 1 | 8,5 | 39,3 | 49,2 | 1 | 2464,91 | 2800,64 | 335,73 |
| 3234 | 1 | 0 | 1 | 7,91 | 29,1 | 35,05 | 1 | 1990,86 | 2200,31 | 209,44 |
| 3242 | 1 | 0 | 1 | 8,88 | 35,2 | 47,6 | 1 | 2247,68 | 2724,22 | 476,55 |
| 3284 | 1 | 0 | 2 | 9,37 | 31,6 | 41,2 | 1 | 1860,84 | 2157,36 | 296,52 |
| 3309 | 1 | 0 | 1 | 11,01 | 40,2 | 43,8 | 1 | 2282,43 | 2218,17 | -64,26 |
| 3323 | 1 | 0 | 1 | 10,98 | 45,25 | 53,4 | 1 | 2499,25 | 2620,36 | 121,11 |
| 3328 | 1 | 0 | 2 | 10,07 | 40,3 | 53,1 | 1 | 2162,71 | 2581,02 | 418,31 |
| 3330 | 1 | 0 | 1 | 10,3 | 35,95 | 39,8 | 1 | 2166,11 | 2144,69 | -21,42 |
| 4326 | 1 | 0 | 1 | 6,99 | 23 | 27,35 | 1 | 1703,43 | 1879,90 | 176,47 |
| 5328 | 1 | 0 | 1 | 6,06 | 22,5 | 26,3 | 1 | 1708,83 | 1854,01 | 145,17 |
| 5372 | 1 | 0 | 2 | 7,04 | 39 | 50,1 | 1 | 2359,59 | 2734,83 | 375,25 |
| 5373 | 1 | 0 | 1 | 6,98 | 29,8 | 36,25 | 1 | 2068,27 | 2313,93 | 245,66 |
| 5375 | 1 | 0 | 2 | 6,97 | 24,35 | 30,2 | 1 | 1631,88 | 1857,74 | 225,86 |
| 5378 | 1 | 0 | 2 | 6,82 | 28 | 36,8 | 1 | 1825,28 | 2177,48 | 352,20 |
| 5384 | 1 | 0 | 2 | 6,52 | 24,2 | 29,6 | 1 | 1645,22 | 1846,49 | 201,27 |
| 5386 | 1 | 0 | 1 | 6,19 | 25,45 | 31,05 | 1 | 1876,24 | 2089,81 | 213,57 |
| 5396 | 1 | 0 | 2 | 8,95 | 37,8 | 47,55 | 1 | 2175,63 | 2434,35 | 258,72 |
| 5405 | 1 | 0 | 1 | 8,04 | 29,2 | 35 | 1 | 1990,84 | 2186,22 | 195,38 |
| 5482 | 1 | 0 | 1 | 10,92 | 54,05 | 69,3 | 1 | 2865,75 | 3266,09 | 400,34 |
| 5491 | 1 | 0 | 1 | 10,29 | 39,3 | 52,3 | 1 | 2317,94 | 2735,62 | 417,68 |
| 5494 | 1 | 0 | 2 | 10,18 | 43,55 | 54,2 | 1 | 2286,08 | 2577,49 | 291,41 |
| 5497 | 1 | 0 | 1 | 9,86 | 37,6 | 44,1 | 1 | 2281,72 | 2400,36 | 118,64 |
| 5508 | 1 | 0 | 1 | 10,23 | 43 | 52 | 1 | 2487,81 | 2682,47 | 194,66 |
| 5510 | 1 | 0 | 2 | 10,18 | 45,25 | 51,3 | 1 | 2355,68 | 2414,24 | 58,57 |
| 5523 | 1 | 0 | 1 | 9,79 | 37,65 | 44,95 | 1 | 2290,85 | 2451,98 | 161,13 |
| 5579 | 1 | 0 | 2 | 9,05 | 32,75 | 45,2 | 1 | 1941,69 | 2368,16 | 426,47 |
| 5584 | 1 | 0 | 2 | 9,81 | 33,45 | 41,8 | 1 | 1897,57 | 2131,60 | 234,03 |
| 5591 | 1 | 0 | 2 | 9,21 | 37,25 | 46 | 1 | 2127,86 | 2342,95 | 215,09 |
| 5592 | 1 | 0 | 1 | 9,77 | 44,8 | 51,25 | 1 | 2615,71 | 2686,98 | 71,27 |
| 5602 | 1 | 0 | 2 | 11,44 | 45,2 | 56 | 1 | 2232,12 | 2522,81 | 290,69 |
| 5608 | 1 | 0 | 2 | 8,82 | 43,3 | 52,3 | 1 | 2426,18 | 2614,67 | 188,49 |
| 5612 | 1 | 0 | 2 | 8,82 | 36,6 | 46,15 | 1 | 2131,59 | 2389,36 | 257,77 |
| 5617 | 1 | 0 | 1 | 11,34 | 40,1 | 45,1 | 1 | 2237,86 | 2249,34 | 11,48 |
| 5620 | 1 | 0 | 1 | 8,96 | 49,8 | 55,8 | 1 | 2924,93 | 2956,49 | 31,56 |
| 5629 | 1 | 0 | 1 | 10,55 | 42,35 | 48,35 | 1 | 2425,26 | 2467,02 | 41,76 |
| 5633 | 1 | 0 | 1 | 6,48 | 23,45 | 28 | 1 | 1742,65 | 1931,41 | 188,77 |
| 5638 | 1 | 0 | 2 | 7,03 | 34,95 | 43,8 | 1 | 2158,10 | 2457,14 | 299,04 |
| 5640 | 1 | 0 | 1 | 8,13 | 32,2 | 35,5 | 1 | 2137,98 | 2177,41 | 39,43 |
| 5646 | 1 | 0 | 1 | 8,02 | 31,4 | 36,55 | 1 | 2102,87 | 2249,54 | 146,67 |
| 5647 | 1 | 0 | 1 | 6,48 | 24,1 | 30,2 | 1 | 1782,83 | 2047,47 | 264,64 |
| 5649 | 1 | 0 | 2 | 7,9 | 44,6 | 54,4 | 1 | 2552,88 | 2803,79 | 250,91 |
| 5736 | 1 | 0 | 2 | 7,69 | 32 | 40 | 1 | 1977,12 | 2241,79 | 264,67 |
| 5943 | 1 | 0 | 1 | 7,9 | 27,6 | 32 | 1 | 1914,38 | 2045,91 | 131,53 |
| 5953 | 1 | 0 | 2 | 6,38 | 27,65 | 31,7 | 1 | 1838,71 | 1929,73 | 91,02 |
| 5954 | 1 | 0 | 2 | 8,7 | 38,7 | 44,7 | 1 | 2233,29 | 2296,88 | 63,59 |
| 5958 | 1 | 0 | 2 | 10,61 | 44 | 55,35 | 1 | 2259,87 | 2583,09 | 323,22 |
| 5987 | 1 | 0 | 1 | 9,95 | 43 | 47,15 | 1 | 2516,64 | 2472,96 | -43,68 |
| 1104 | 1 | 1 | 1 | 8,51 | 38,5 | 56,3 | 1 | 2426,21 | 3188,69 | 762,48 |
| 1117 | 1 | 1 | 1 | 9,21 | 31,25 | 34,75 | 1 | 2031,47 | 2050,11 | 18,64 |
| 1137 | 1 | 1 | 1 | 6,1 | 24,75 | 32,2 | 1 | 1842,12 | 2164,07 | 321,96 |
| 1185 | 1 | 1 | 1 | 6,6 | 33 | 53,05 | 1 | 2264,98 | 3220,58 | 955,60 |
| 1219 | 1 | 1 | 1 | 6,96 | 32,1 | 42,85 | 1 | 2190,95 | 2653,21 | 462,26 |
| 1220 | 1 | 1 | 2 | 7,26 | 27 | 31,9 | 1 | 1752,49 | 1901,52 | 149,03 |
| 1240 | 1 | 1 | 2 | 6,81 | 24,8 | 29,6 | 1 | 1661,70 | 1828,16 | 166,46 |
| 1260 | 1 | 1 | 2 | 8,36 | 35,9 | 49,4 | 1 | 2127,64 | 2608,97 | 481,33 |
| 1273 | 1 | 1 | 1 | 8,24 | 37 | 44,9 | 1 | 2368,76 | 2626,19 | 257,43 |
| 1350 | 1 | 1 | 1 | 10,16 | 39,5 | 46,2 | 1 | 2339,55 | 2445,96 | 106,41 |
| 1358 | 1 | 1 | 2 | 10,6 | 42,2 | 55 | 1 | 2188,14 | 2595,13 | 406,99 |
| 1475 | 1 | 1 | 1 | 6,98 | 22,65 | 27,2 | 1 | 1684,97 | 1874,33 | 189,37 |
| 1513 | 1 | 1 | 1 | 6,96 | 25,25 | 30,45 | 1 | 1826,03 | 2035,13 | 209,10 |
| 1516 | 1 | 1 | 1 | 6,37 | 28,1 | 36,1 | 1 | 2013,80 | 2343,98 | 330,18 |
| 1522 | 1 | 1 | 2 | 6,83 | 27,5 | 36,7 | 1 | 1799,13 | 2175,68 | 376,55 |
| 1557 | 1 | 1 | 1 | 7,94 | 41 | 55,6 | 1 | 2584,09 | 3182,73 | 598,64 |
| 1560 | 1 | 1 | 2 | 7,29 | 40,3 | 43,3 | 1 | 2399,34 | 2347,80 | -51,54 |
| 1595 | 1 | 1 | 1 | 7,65 | 29,9 | 33,6 | 1 | 2042,06 | 2132,97 | 90,91 |
| 1605 | 1 | 1 | 1 | 8,92 | 36,1 | 40,6 | 1 | 2288,65 | 2335,80 | 47,14 |
| 1606 | 1 | 1 | 1 | 7,48 | 22,6 | 26,6 | 1 | 1666,05 | 1819,78 | 153,74 |
| 1611 | 1 | 1 | 2 | 7,17 | 35,1 | 47,55 | 1 | 2154,69 | 2641,50 | 486,81 |
| 1632 | 1 | 1 | 1 | 8,92 | 32,05 | 39,2 | 1 | 2092,92 | 2307,53 | 214,61 |
| 1693 | 1 | 1 | 2 | 9,58 | 38,4 | 50,45 | 1 | 2138,71 | 2522,09 | 383,39 |
| 1701 | 1 | 1 | 1 | 9,53 | 39,3 | 51 | 1 | 2390,98 | 2778,79 | 387,81 |
| 1707 | 1 | 1 | 1 | 9,41 | 38,05 | 48,95 | 1 | 2343,65 | 2702,76 | 359,11 |
| 1725 | 1 | 1 | 2 | 8,98 | 39,55 | 54,85 | 1 | 2250,33 | 2778,51 | 528,18 |
| 1755 | 1 | 1 | 2 | 10,01 | 37,1 | 46,85 | 1 | 2033,28 | 2318,79 | 285,51 |
| 1765 | 1 | 1 | 1 | 10,04 | 32,8 | 36,95 | 1 | 2042,47 | 2064,85 | 22,38 |
| 1780 | 1 | 1 | 2 | 10,97 | 50 | 59,8 | 1 | 2461,67 | 2674,43 | 212,76 |
| 1796 | 1 | 1 | 2 | 10,68 | 36,05 | 43,4 | 1 | 1927,73 | 2113,62 | 185,89 |
| 1801 | 1 | 1 | 2 | 10,98 | 42,9 | 58,15 | 1 | 2183,36 | 2697,46 | 514,09 |
| 1807 | 1 | 1 | 2 | 10,53 | 37,5 | 56,4 | 1 | 1999,99 | 2729,10 | 729,11 |
| 2091 | 1 | 1 | 1 | 6,14 | 26,5 | 36,2 | 1 | 1941,13 | 2368,77 | 427,64 |
| 2098 | 1 | 1 | 2 | 7,04 | 23 | 29,35 | 1 | 1559,36 | 1820,72 | 261,36 |
| 2109 | 1 | 1 | 1 | 7,59 | 22,7 | 27,7 | 1 | 1667,82 | 1872,63 | 204,81 |
| 2118 | 1 | 1 | 1 | 6,81 | 30 | 38,2 | 1 | 2088,33 | 2427,13 | 338,80 |
| 2148 | 1 | 1 | 2 | 8,35 | 31,25 | 37,9 | 1 | 1914,04 | 2070,95 | 156,92 |
| 2152 | 1 | 1 | 1 | 9,39 | 31,2 | 36,75 | 1 | 2018,21 | 2143,20 | 124,99 |
| 2165 | 1 | 1 | 1 | 9,76 | 42,65 | 49,3 | 1 | 2521,05 | 2617,35 | 96,30 |
| 2190 | 1 | 1 | 1 | 10,55 | 55,7 | 68,8 | 1 | 2984,07 | 3278,34 | 294,27 |
| 2201 | 1 | 1 | 1 | 11,48 | 64,5 | 78,35 | 1 | 3178,59 | 3439,75 | 261,17 |
| 2206 | 1 | 1 | 1 | 10,11 | 40,6 | 49,1 | 1 | 2393,52 | 2584,06 | 190,54 |
| 2564 | 1 | 1 | 1 | 6,59 | 22,55 | 26 | 1 | 1691,40 | 1822,66 | 131,26 |
| 2600 | 1 | 1 | 2 | 7,15 | 26,3 | 34,05 | 1 | 1722,43 | 2031,94 | 309,51 |
| 2628 | 1 | 1 | 2 | 8,04 | 28,35 | 34,05 | 1 | 1790,32 | 1932,05 | 141,73 |
| 2648 | 1 | 1 | 2 | 8,51 | 32,7 | 39,8 | 1 | 1973,28 | 2137,11 | 163,83 |
| 2685 | 1 | 1 | 2 | 9,46 | 33 | 40,45 | 1 | 1913,64 | 2098,29 | 184,65 |
| 2689 | 1 | 1 | 2 | 9,43 | 38,95 | 49,3 | 1 | 2178,97 | 2467,79 | 288,81 |
| 2693 | 1 | 1 | 2 | 9,62 | 36,7 | 46,8 | 1 | 2060,44 | 2354,40 | 293,96 |
| 2704 | 1 | 1 | 2 | 10,27 | 44,25 | 51,35 | 1 | 2305,01 | 2423,50 | 118,48 |
| 2719 | 1 | 1 | 1 | 10,18 | 46,55 | 54,8 | 1 | 2647,78 | 2783,00 | 135,23 |
| 2722 | 1 | 1 | 1 | 11,6 | 39,7 | 50,3 | 1 | 2190,03 | 2474,14 | 284,11 |
| 3025 | 1 | 1 | 1 | 9,27 | 40,3 | 45,4 | 1 | 2459,88 | 2504,83 | 44,95 |
| 3118 | 1 | 1 | 2 | 6,48 | 30,55 | 39,1 | 1 | 1983,20 | 2285,48 | 302,28 |
| 3137 | 1 | 1 | 1 | 6,51 | 22,95 | 28,5 | 1 | 1715,36 | 1960,62 | 245,26 |
| 3147 | 1 | 1 | 2 | 6,5 | 35,25 | 50,6 | 1 | 2225,99 | 2832,88 | 606,89 |
| 3154 | 1 | 1 | 1 | 6,46 | 30,6 | 38,3 | 1 | 2145,88 | 2437,43 | 291,56 |
| 3166 | 1 | 1 | 1 | 7,86 | 26 | 35,8 | 1 | 1831,34 | 2272,33 | 440,99 |
| 3204 | 1 | 1 | 2 | 7,71 | 32,4 | 39,4 | 1 | 1995,31 | 2202,55 | 207,24 |
| 3205 | 1 | 1 | 2 | 7,42 | 33,7 | 42,3 | 1 | 2071,47 | 2367,26 | 295,79 |
| 3245 | 1 | 1 | 1 | 8,75 | 41,4 | 55,4 | 1 | 2548,99 | 3079,26 | 530,28 |
| 3248 | 1 | 1 | 2 | 8,56 | 34,95 | 45,8 | 1 | 2073,48 | 2412,72 | 339,25 |
| 3264 | 1 | 1 | 1 | 8,96 | 34,3 | 40,4 | 1 | 2199,91 | 2344,48 | 144,56 |
| 3274 | 1 | 1 | 2 | 9,34 | 38,2 | 46,45 | 1 | 2156,10 | 2344,48 | 188,38 |
| 3276 | 1 | 1 | 2 | 9,1 | 46,5 | 61,8 | 1 | 2537,72 | 3020,91 | 483,19 |
| 3295 | 1 | 1 | 1 | 9,9 | 33,65 | 39,7 | 1 | 2091,96 | 2209,88 | 117,92 |
| 3311 | 1 | 1 | 1 | 10,22 | 42,3 | 48,9 | 1 | 2458,16 | 2538,54 | 80,38 |
| 5342 | 1 | 1 | 1 | 7,15 | 29,65 | 37,05 | 1 | 2051,52 | 2352,51 | 300,99 |
| 5351 | 1 | 1 | 2 | 6,8 | 23,8 | 29,3 | 1 | 1605,64 | 1824,19 | 218,55 |
| 5352 | 1 | 1 | 1 | 6,76 | 28,5 | 37,1 | 1 | 2010,56 | 2381,27 | 370,70 |
| 5368 | 1 | 1 | 1 | 7,1 | 35,35 | 45,25 | 1 | 2350,55 | 2747,10 | 396,55 |
| 5380 | 1 | 1 | 1 | 6,73 | 27,45 | 37,7 | 1 | 1955,24 | 2425,95 | 470,71 |
| 5383 | 1 | 1 | 2 | 6,54 | 27,75 | 33,4 | 1 | 1831,05 | 2009,15 | 178,10 |
| 5395 | 1 | 1 | 1 | 8,98 | 33,4 | 40,7 | 1 | 2155,36 | 2367,74 | 212,37 |
| 5399 | 1 | 1 | 1 | 8,73 | 33,3 | 41,2 | 1 | 2163,65 | 2418,60 | 254,96 |
| 5407 | 1 | 1 | 2 | 7,9 | 42,7 | 50,7 | 1 | 2466,31 | 2637,12 | 170,82 |
| 5409 | 1 | 1 | 2 | 7,85 | 30,6 | 37,5 | 1 | 1904,24 | 2109,61 | 205,37 |
| 5425 | 1 | 1 | 2 | 7,9 | 37,2 | 48,5 | 1 | 2210,94 | 2599,56 | 388,62 |
| 5435 | 1 | 1 | 1 | 7,63 | 29,25 | 38,5 | 1 | 2009,44 | 2403,59 | 394,15 |
| 5438 | 1 | 1 | 1 | 7,5 | 32,2 | 42,6 | 1 | 2166,37 | 2609,14 | 442,77 |
| 5442 | 1 | 1 | 2 | 9,42 | 50 | 61,1 | 1 | 2646,44 | 2902,85 | 256,41 |
| 5448 | 1 | 1 | 2 | 9,04 | 36,25 | 44,3 | 1 | 2099,52 | 2282,79 | 183,27 |
| 5455 | 1 | 1 | 2 | 8,93 | 29,7 | 36,85 | 1 | 1811,08 | 1986,42 | 175,34 |
| 5531 | 1 | 1 | 2 | 8,96 | 37,25 | 45 | 1 | 2150,44 | 2312,15 | 161,71 |
| 5535 | 1 | 1 | 1 | 11,63 | 50,4 | 61,6 | 1 | 2617,54 | 2862,04 | 244,49 |
| 5537 | 1 | 1 | 2 | 11,42 | 47,45 | 61,8 | 1 | 2320,01 | 2762,44 | 442,43 |
| 5569 | 1 | 1 | 1 | 9,33 | 34,6 | 43,5 | 1 | 2187,65 | 2463,65 | 276,00 |
| 5573 | 1 | 1 | 2 | 10,54 | 31,8 | 48,1 | 1 | 1758,40 | 2397,38 | 638,98 |
| 5586 | 1 | 1 | 1 | 11,45 | 46,55 | 62,15 | 1 | 2489,34 | 2961,79 | 472,44 |
| 5589 | 1 | 1 | 2 | 10,52 | 51,2 | 61,55 | 1 | 2554,65 | 2785,07 | 230,42 |
| 5596 | 1 | 1 | 2 | 10,36 | 45,9 | 52,5 | 1 | 2362,20 | 2446,39 | 84,20 |
| 5600 | 1 | 1 | 1 | 9,29 | 42 | 54,9 | 1 | 2536,37 | 2978,61 | 442,24 |
| 5610 | 1 | 1 | 2 | 11,05 | 42,5 | 50,3 | 1 | 2161,32 | 2331,04 | 169,72 |
| 5614 | 1 | 1 | 1 | 10,61 | 35,9 | 41,45 | 1 | 2133,11 | 2196,82 | 63,71 |
| 5623 | 1 | 1 | 1 | 9,38 | 42,4 | 51,15 | 1 | 2546,55 | 2767,24 | 220,69 |
| 5624 | 1 | 1 | 2 | 6,38 | 27 | 34,5 | 1 | 1803,90 | 2082,99 | 279,09 |
| 5635 | 1 | 1 | 2 | 6,3 | 22,5 | 25,3 | 1 | 1562,57 | 1635,59 | 73,02 |
| 5643 | 1 | 1 | 2 | 7,56 | 52,05 | 62,25 | 1 | 2922,02 | 3137,35 | 215,34 |
| 5644 | 1 | 1 | 1 | 6,5 | 24,15 | 28,8 | 1 | 1784,75 | 1968,85 | 184,10 |
| 5657 | 1 | 1 | 2 | 6,93 | 25,7 | 29,65 | 1 | 1702,37 | 1818,85 | 116,48 |
| 5944 | 1 | 1 | 2 | 8,01 | 38,9 | 49,4 | 1 | 2283,26 | 2609,71 | 326,45 |
| 5988 | 1 | 1 | 1 | 10,26 | 40,1 | 51,1 | 1 | 2356,58 | 2670,50 | 313,92 |

References

1. Schonbeck Y, Talma H, van Dommelen P, Bakker B, Buitendijk SE, Hirasing RA, et al. Increase in prevalence of overweight in Dutch children and adolescents: a comparison of nationwide growth studies in 1980, 1997 and 2009. PLoS One. 2011;6: e27608.

2. Cole TJ, Bellizzi MC, Flegal KM, Dietz WH. Establishing a standard definition for child overweight and obesity worldwide: international survey. Bmj. 2000;320: 1240-1243.

3. Cole TJ, Flegal KM, Nicholls D, Jackson AA. Body mass index cut offs to define thinness in children and adolescents: international survey. Bmj. 2007;335: 194.

4. Hall KD, Butte NF, Swinburn BA, Chow CC. Dynamics of childhood growth and obesity: development and validation of a quantitative mathematical model. Lancet Diabetes Endocrinol. 2013;1: 97-105.
