# Supplementary material for: Impact of Masked Replacement of Sugar-Sweetened with Sugar-Free Beverages on Body Weight Increases with Initial BMI: Secondary Analysis of Data from an 18 Month Double–Blind Trial in Children
Source: PLoS One. 2016 Jul 22;11(7):e0159771. doi: 10.1371/journal.pone.0159771 (PMC4957753; doi:10.1371/journal.pone.0159771)
Supplement: S1 Protocol — (PDF) [file pone.0159771.s004.pdf]

RESEARCH PROTOCOL

A DOUBLE-BLIND RANDOMIZED TRIAL OF THE EFFICACY OF REPLACING SUGARY DRINKS BY LOW SUGAR ALTERNATIVES ON BODY WEIGHT AND FAT MASS IN SCHOOL CHILDREN

(Feb. 2009)

A double-blind randomized trial of the efficacy of replacing sugary drinks by low-sugar alternatives on body weight and fat mass in school children

Protocol ID	120520010 (ZonMw)
Short title	DRINK-study
Version	1
Date	03-02-2009
Coordinating investigator/project leader	Prof. dr. Martijn B. Katan<br>Vrije Universiteit<br>Faculty of Earth and Life Sciences<br>Institute of Health Sciences<br>De Boelelaan 1085<br>1081 HV Amsterdam<br>ph. (GSM): +31 (0) 654 283 500
Principal investigator(s) (in Dutch: hoofdonderzoeker/uitvoerder)<br>*Multicenter research: per site*	Dr. ir. Margreet R. Olthof<br>Vrije Universiteit<br>Faculty of Earth and Life Sciences<br>Institute of Health Sciences<br>De Boelelaan 1085<br>1081 HV Amsterdam<br>The Netherlands<br>ph. +31 (0)20 5982522
Sponsor (in Dutch: verrichter/opdrachtgever)<br><br><br>Subsidising parties	Prof. dr. Martijn B. Katan<br>Institute of Health Sciences<br>VU University<br><br>ZonMw<br>Nederlandse Hartstichting<br>KNAW
Sponsor	The sponsor is the party that commissions the organisation or performance of the research, for example a pharmaceutical company, academic hospital, scientific organisation or investigator. A party that provides funding for a study but does not commission it is not regarded as the sponsor, but

	referred to as a subsidising party.
Independent physician	Ellinore Tellegen<br>General practitioner, Purmerend<br>Tel: 0299-644011
Laboratory sites	Not applicable
Pharmacy	Not applicable

PROTOCOL SIGNATURE SHEET

Name	Signature	Date
For non-commercial research,<br>Head of Department:	Prof. dr. J.C. Seidell<br>*Professor of Nutrition and Health, Director Institute for Health Sciences*<br>*Vrije Universiteit and VU Medical Center, Amsterdam*	
Coordinating Investigator/Project leader:	Prof. Dr. Martijn B. Katan<br>*Project leader*	
Principal Investigator	Dr. Ir. Margreet R. Olthof<br>*Principal investigator*	

TABLE OF CONTENTS

SUMMARY 9
1. INTRODUCTION AND RATIONALE 11
2. OBJECTIVES 13
3. STUDY DESIGN 14
4. STUDY POPULATION 17
4.1 Population (base) 17
4.2 Inclusion criteria 17
4.3 Exclusion criteria 17
4.4 Sample size calculation 18
5. TREATMENT OF SUBJECTS 19
5.1 Investigational product/treatment 19
5.2 Sweeteners 20
5.3 Use of co-intervention (if applicable) 21
5.4 Escape medication (if applicable) 21
6. INVESTIGATIONAL MEDICINAL PRODUCT 22
7. METHODS 23
7.1 Study parameters/endpoints 23
7.1.01 Main study parameter/endpoint 23
7.1.02 Secondary study parameters/endpoints 23
7.1.03 Other study parameters 23
7.2 Randomisation, blinding and treatment allocation 23
7.3 Study procedures 24
7.4 Withdrawal of individual subjects 25
7.4.01 Specific criteria for withdrawal 25
7.5 Replacement of individual subjects after withdrawal 25
7.6 Follow-up of subjects withdrawn from treatment 25
7.7 Premature termination of the study 25
8. SAFETY REPORTING 26
8.1 Section 10 WMO event 26
8.2 Adverse and serious adverse events 26
8.2.01 Suspected unexpected serious adverse reactions (SUSAR) 26
8.2.02 Annual safety report 26
8.3 Follow-up of adverse events 27
8.4 Data Safety Monitoring Board (DSMB) 27
9. STATISTICAL ANALYSIS 28
9.1 Descriptive statistics 28
9.2 Univariate analysis 28
9.3 Multivariate analysis 28
9.4 Interim analysis 28
10. ETHICAL CONSIDERATIONS 29
10.1 Regulation statement 29

10.2 Recruitment and consent ... 29
10.3 Objection by minors or incapacitated subjects (if applicable) ... 29
10.4 Benefits and risks assessment, group relatedness ... 30
10.5 Compensation for injury ... 30
10.6 Incentives ... 31
11. ADMINISTRATIVE ASPECTS AND PUBLICATION ... 32
11.1 Handling and storage of data and documents ... 32
11.2 Amendments ... 32
11.3 Annual progress report ... 32
11.4 End of study report ... 32
11.5 Public disclosure and publication policy ... 33
REFERENCES ... 34

Amendement 1 ... 35
Amendement 2 ... 36
Amendement 3 ... 38

LIST OF ABBREVIATIONS AND RELEVANT DEFINITIONS

ABR	ABR form (General Assessment and Registration form) is the application form that is required for submission to the accredited Ethics Committee (ABR = Algemene Beoordeling en Registratie)
AE	Adverse Event
AR	Adverse Reaction
CA	Competent Authority
CCMO	Central Committee on Research Involving Human Subjects
CV	Curriculum Vitae
DSMB	Data Safety Monitoring Board
EU	European Union
EudraCT	European drug regulatory affairs Clinical Trials GCP Good Clinical Practice
IB	Investigator's Brochure
IC	Informed Consent
IMP	Investigational Medicinal Product
IMPD	Investigational Medicinal Product Dossier
METC	Medical research ethics committee (MREC); in Dutch: medisch ethische toetsing commissie (METC)
(S)AE	Serious Adverse Event
SPC	Summary of Product Characteristics (in Dutch: officiële productinfomatie IB1-tekst)
Sponsor	The sponsor is the party that commissions the organisation or performance of the research, for example a pharmaceutical company, academic hospital, scientific organisation or investigator. A party that provides funding for a study but does not commission it is not regarded as the sponsor, but referred to as a subsidising party.
SUSAR	Suspected Unexpected Serious Adverse Reaction
Wbp	Personal Data Protection Act (in Dutch: Wet Bescherming Persoonsgevens)
WMO	Medical Research Involving Human Subjects Act (Wet Medisch-wetenschappelijk Onderzoek met Mensen

SUMMARY

Rationale: Liquid carbohydrates (including soft drinks as well as fruit juices) are thought to be less satiating then solid carbohydrates (e.g. bread or fruits). Calories from sugary drinks might not be compensated for by eating less at subsequent meals. In this way liquid carbohydrates might be one of the causes of overweight and obesity in children. However government policies to reduce intake have been less than firm. One reason may be that the evidence for an effect of sugary drinks on body weight rests mostly on observational epidemiological studies and a few short term interventions. Therefore, we propose the first double-blind, long term, randomized controlled trial. The hypothesis of this trial is that intake of liquid carbohydrates is not compensated for sufficiently by reducing caloric consumption for other foods. This leads to incomplete compensation for the energy ingested and eventually results in the excess weight gain.
Objective: To test the effect of replacing sugar containing beverages by 'diet' beverages containing non-caloric sweeteners on body weight and fat mass in school children aged 5-10 years. In addition we will examine the food intake of all participants at lunch time. This trial is a strict test of the physiological effects of liquid carbohydrates on body weight. Changes in body weight due to social and psychological cues and expectations will be eliminated since the trial is blinded.
Study design: A double-blind, long term, randomized controlled trial
Study population: Healthy school children in the age of 5-10 years old. We consider it unethical to encourage children to drink sugary drinks. Therefore children are only eligible if they already habitually consume 250 mL per day or more of sugary drinks.
Intervention: Six hundred healthy children (5-10 years) will be divided randomly into 2 groups. Group 1 (n=300) receives 250 mL per day of sugar-containing drinks (Unilever peach drink, Unilever mango drink, Unilever lemon drink). Group 2 (n=300) receive 250 mL per day of light drinks (Unilever peach drink light, Unilever mango drink light, Unilever lemon drink light). The drinks for group 2 are identical looking. The light drinks are sweetened with artificial sweeteners. The drinks will be consumed during the morning break during the weekdays at school and at home during weekends and holidays. The intervention period for both groups will be 18 months.
Main study parameters/endpoints: The primary outcomes of the study are the children's bodyweight (body mass index, corrected for age), waist circumference, skin folds and bioelectrical impedance. These outcomes will be measured four times during the study, at the start, after 6, 12 months and at the end of the study. As a secondary outcome we will also assess food intake of the children at lunch time, shortly after the morning break when

the children have consumed the study drinks. This outcome will be measured at the start and at the end of the study.

Nature and extent of the burden and risks associated with participation, benefit and group relatedness: All drinks are safe. The amounts of artificial sweeteners in the light drinks are in accordance with governmental regulations for foods and drinks (http://www.voedingscentrum.nl). The measurements are non-invasive and there are no risks associated to these measurements. Measurements will be done by experienced and trained researchers. There is no direct clinical benefit from participating in this study. However this trial might have a decisive impact on policy and medical practise and lead to effective interventions that contribute to the prevention of overweight and obesity in children.

1. INTRODUCTION AND RATIONALE

The prevalence of childhood obesity is rising rapidly (1). Obese children often become obese adults (2) with the associated risks of type 2 diabetes, cancer and cardiovascular diseases (3-6). In addition, children with overweight are often stigmatized and bullied by other children (7; 8). In 2002-2004 about 15% of Dutch children between 4 and 15 years old were overweight and 3% were obese and this is expected to rise (9). Thus prevention of obesity at a young age is crucial to prevent chronic diseases both in childhood and in adult life. Obesity results from an imbalance between energy intake and energy expenditure. There is much speculation about foods that are particularly fattening, and soft drinks are seen as major culprits (13). This belief already forms part of government nutrition policy, but surprisingly, evidence from properly conducted clinical trials is almost nonexistent. It has been postulated that liquid carbohydrates are less satiating than solid carbohydrates, and that calories from sugary drinks are invisible to the system that regulates food intake. As a result, a high intake of sugary drinks would not be compensated by reduced food intake at subsequent meals and body weight would increase. DiMeglio and Mattes (14) showed that consumption of 450 kcal soda/d during 4 weeks resulted in a small increase in body weight relative to consumption of an isocaloric solid carbohydrate load. However, this study was short term, not blinded, and performed in adults.

The evidence for a causal relation between sugar-sweetened beverages and weight gain is inconclusive (13). Observational studies indeed suggest a positive association (13), but people who drink more soft drinks often eat more fast food, exercise less, and care less about their weight. Such confounders are difficult to eliminate completely. Some trials in adults (14-16) support the hypothesis that intake of sugar-sweetened beverages causes weight gain, but these studies were small, short term, and not properly blinded. Two intervention studies in children produced equivocal results. James et al. (17) found that a school-based educational program aimed to reduce consumption of carbonated drinks did not affect body mass index, although the authors did claim that it reduced the proportion of overweight and obese children. Ebbeling et al. (18) found no significant effect on body mass index of providing sugar-free beverages to teenagers. However, in a post-hoc analysis the change in BMI did differ significantly between intervention and control in the upper tertile of baseline BMI. These studies have serious shortcomings, such as no proper blinding, no proper placebo treatment, small samples sizes, and short duration. Therefore, the main objective of our research proposal is to do the first large, double blinded, long-term, controlled trial of the effects of sugar-sweetened beverages on body weight in children. We aim at children because obesity starts in childhood, and once it is present it is hard to rectify. Also, children are less weight-conscious than adults, and therefore they are less likely to

change their food intake or level of exercise when they gain or lose body weight. We consider it unethical to encourage children to drink sugary drinks. Therefore, children are only eligible if they already habitually consume more than 250 mL of sugar-sweetened beverages per day. This way, children will replace their habitual drinks with the study drinks, without a need to change their drinking habits. If sugar sweetened beverages are conclusively shown to cause weight gain, public health strategies to discourage consumption of sugary drinks should be implemented more vigorously. However, if no effect of sugar sweetened beverages on weight gain is seen in a properly designed and powered trial then other, more effective interventions to decrease overweight in children should be pursued and implemented. In summary:

-Overweight in children is an increasing and health-threatening problem.

-Sugary drinks are viewed as a major cause.

-Policies to reduce intake have been hesitant, in part because solid evidence from long term, double-blinded intervention trials is lacking.

2. OBJECTIVES

The following research questions have been defined:

Primary objective:

- What is the effect of replacing beverages with sugar by low sugar alternatives on body weight and fat mass in school children aged 5-10 years old.

Secondary objective:

- What is the effect of replacing beverages with sugar by low sugar alternatives on food intake at lunch time shortly after the morning break when the school children aged 5-10 years old have consumed the study drinks?

3. STUDY DESIGN

We will conduct a double-blind, long term, randomised controlled trial with two treatment arms:
1) Sugar containing beverages (for detailed information about the drinks see chapter 5)
2) Beverages without sugar and with artificial sweeteners (for detailed information about the drinks see chapter 5).

(several flavours of the drinks will be available so children can choose and vary the flavours during the study)

Children will be randomly assigned to one of the two treatments. The treatments will be double blinded. The duration of the trial will be 18 months and will be executed at primary schools. During summer holidays children will not receive the study drinks because of logistic reasons. This time will be added at the end of the intervention. Note that the total intervention time will stay 18 months. Six hundred children will participate in this study and we want as many children per school as possible to minimise the number of participating primary schools. During the trial drinks will be consumed during the morning break at school and at home during weekends and holidays. The teachers at the school will ensure that the children drink their own study drink and that they do not consume drinks from home. In weekends and holidays parents will be asked to ensure that children consume the drinks as a replacement of the drinks that they would receive normally. Parents will be asked to offer the drink around the same time as the morning break to set up a good routine for children. Before we start the trial, a pilot study of two months will be done at one primary school. Around 80 children will participate in the pilot study.

<u>Restrictions during the pilot study/ main trial</u>
The main instruction for the participants of the pilot study/ main trial is to consume the study drink. No other instructions will be given in order to maintain spontaneous food intake and behavior. In this way the only difference between the two intervention groups will be the study drink they consume. We expect that changes in dietary or habits will occur equally in both groups. During the whole study period parents will fill in a diary to inform the researchers about specific things such as when children have not consumed the (whole) study drink, illness and use of medicines.

<u>PILOT STUDY:</u>

Before the main trial starts we will test feasibility and logistics in a pilot study. Two of the drinks that will be used in the trial will be used for the pilot study: Unilever peach drink and Unilever lemon drink. The aims of the pilot study are:
-To test whether the planned study drinks (regular and light) are acceptable for the children
-To test compliance at school during the week days and at home during weekends and holidays (teachers observation and checking ingestion of the study drinks by the children through collecting empty packages).
-To test logistics of beverage production, packaging, coding, transport and storage
-To test feasibility of measuring weight, height, skin folds, bioelectrical impedance and waist circumference at the schools
The design of the pilot study will be a 2-month randomized controlled trial in approximately 80 school children aged 5-10 years. The following measurements will be done at the beginning and the end of the pilot study: weight, height, waist circumference (cm), skin fold thickness (mm) and bioelectrical impedance. Skin fold thickness will be measured at the biceps and triceps.

TIMING OF THE MEASUREMENTS IN PILOT STUDY:

Measurement 1: start of the pilot:

- Weight and height for calculation of Body mass index (corrected for age)
- Fat mass as estimated from waist circumference (cm), skin fold thickness (mm) and bioelectrical impedance. Skin fold thickness will be measured at the biceps and triceps.

Measurement 2: end of the pilot (after 2 months)

- Weight and height for calculation of Body mass index (corrected for age)
- Fat mass as estimated from waist circumference (cm), skin fold thickness (mm) and body impedance. Skin fold thickness will be measured at the biceps and triceps.

MAIN TRIAL:

During the trial the following measurements will be done at the beginning and after 6, 12 and 18 months: body weight and height for calculation of Body mass index (corrected for age). Fat mass will be estimated from waist circumference (cm), skin fold thickness (mm) and bioelectrical impedance. Skin fold thickness will be measured at the biceps and triceps. At the beginning and the end of the trial we will measure energy intake at lunch on one day.

TIMING OF THE MEASUREMENTS IN MAIN TRIAL:

Measurement 1: start of the trial

- Weight and height for calculation of Body mass index (corrected for age)

- Fat mass as estimated from waist circumference (cm) and skin fold thickness (mm) and bioelectrical impedance. Skin fold thickness will be measured at the biceps and triceps
- Food intake at lunch time

<u>Measurement 2: after 6 months</u>

- Weight and height for calculation of Body mass index (corrected for age)
- Fat mass as estimated from waist circumference (cm), skin fold thickness (mm) and bioelectrical impedance. Skin fold thickness will be measured at the biceps and triceps

<u>Measurement 3: after 12 months</u>

- Weight and height for calculation of Body mass index (corrected for age)
- Fat mass as estimated from waist circumference (cm), skin fold thickness (mm) and bioelectrical impedance. Skin fold thickness will be measured at the biceps and triceps.

<u>Measurement 4: end of the trial (after 18 months)</u>

- Weight and height for calculation of Body mass index (corrected for age)
- Fat mass as estimated from waist circumference (cm) and skin fold thickness (mm) and bioelectrical impedance. Skin fold thickness will be measured at the biceps and triceps
- Food intake at lunchtime

4. STUDY POPULATION

4.1 Population (base)

For the pilot study we will recruit around 80 healthy school going children in the age of 5-10 years old in 1 primary school. For the trial we will recruit 600 healthy school going children in the age of 5-10 years old. We expect that approximately 12 schools will have to participate to find 600 participants for the trial. We consider it unethical to encourage children to drink sugary drinks. Therefore, children are only eligible to participate in the pilot study/ main trial if they already habitually consume more at least 250 mL of sugar-sweetened beverages (e.g. apple juice, orange juice, Wicky, coca cola, milk based drinks such as Fristi, choclate) per day. We aim at children because obesity starts in childhood, and once it is present it is hard to rectify. Also, children are less weight-conscious than adults, and therefore they are less likely to change their food intake or level of exercise when they gain or lose body weight. Schools for the pilot study and the trial will be recruited through collaborations that already exist between our institute and primary schools. Schools will be carefully selected mainly based on the willingness of the school board and teachers to participate in the execution of the pilot study/main trial, i.e. teachers have to be involved in checking whether children ingested the correct (their own) drinks and in collecting empty packages for compliance check. If schools cannot guarantee involvement in the execution of the pilot study/ main trial, they cannot participate. If schools are willing to participate, the parents will be approached through the schools. Parents who are interested to participate will be informed about the pilot study/ main trial orally and in writing by the researchers. Parents will be asked to sign an informed consent form for the participation of his or her child in the pilot study/ main trial separately. Participation is always voluntary and parents may withdraw their child(ren) from the pilot study/ main trial at any point. We expect the recruitment to be feasible since there are many primary schools available that we can approach for participation and collaborations that already exist between our institute and primary schools.

4.2 Inclusion criteria

- Healthy school going boys and girls
- Age 5 years and older, children still have to be in elementary school at the end of the study. .
- Children who already habitually consume 250 mL per day or more of sugary drinks.

4.3 Exclusion criteria

- Using medication or under medical treatment for obesity
- Any acute or chronic disease such as diabetes, grow disorders, celiac disease, or serious gastro-enterology (for example inflammatory bowel disease).

- Medical history or surgical events known to interfere with the study
- Participation in another intervention trial up to 3 months before and during the study if the intervention interferes with the current study
- Physical disabilities that hamper the measurements
- Intention to change location of residence and primary school during the study period

4.4 Sample size calculation

The predicted difference in weight change between the groups is 1.4 kg (21) when 250 mL of the drinks are consumed daily for 1,5 years. This takes into account the increase in metabolic rate when weight increases (21). We assume that at least 70% of the energy from sugar-containing beverages fails to be compensated for by reduced intake of other foods (22). Furthermore, we want to be able to pick up a difference even when compliance is only 80% (i.e. ~200 mL/d of the drinks are actually consumed). This reduces the predicted minimum difference to 750 grams. We therefore need enough power to detect a difference of 750 grams in weight change between the intervention group and the control group after 1,5 years (power = 0.8; alpha = 0.05; SD=2.96 kg (18)). This requires 250 children per treatment group, for a total of 500 children. We expect a drop out of 15%, so we will include 600 children in the study.

5. TREATMENT OF SUBJECTS

5.1 Investigational product/treatment

Participants will be assigned to one out of 2 treatments during the pilot study/ main trial: sugar-containing drinks or low-caloric drinks. The two types of drinks will be produced in various flavours so that children can choose their favourite flavour and can switch flavours when they like. This will increase the compliance. The drinks are designed and will be produced by Unilever and we pay them for doing so. Unilever is no sponsor of this study. As far as we know Unilever will not use these drinks for commercial purposes. L- and A numbers used as ingredients are tested and officially safe ingredients according to the Voedsel and Waren Autoriteit. All drinks will be produced in cans of 250 mL.

A) sugar containing drinks. Flavours: lemon, mango, peach (produced by Unilever)

Ingredients:

1. Lemon flavoured drink (produced by Unilever)

Sugar, lemon flavour (L577), tri-sodium citrate, citric acid, lemon juice, ascorbic acid (commonly known as vitamin c).

2. Peach flavoured drink (produced by Unilever)

Sugar, peach flavour (A676), peach juice, citric acid, ascorbic acid (commonly known as vitamin c).

3. Mango flavoured drink (produced by Unilever)

Sugar, mango flavour (1), mango flavour (2), mango juice, citric acid, ascorbic acid (commonly known as vitamin c).

B) light drinks. Flavours: lemon, mango, peach (produced by Unilever)

Ingredients:

4. Lemon flavoured drink light (produced by Unilever)

Lemon flavour (L577), tri-sodium citrate, citric acid, malic acid, sucralose, acesulfame K, ascorbic acid (commonly known as vitamin c).

5. Peach flavoured drink light (produced by Unilever)

Peach flavour (A676), peach juice, malic acid, tri-sodium citrate, sucralose, acesulfame K, ascorbic acid (commonly known as vitamin c).

6. Mango flavoured drink light (produced by Unilever)

Mango flavour (1), mango flavour (2), mango juice, malic acid, tri-sodium citrate, sucralose, acesulfame K, ascorbic acid (commonly known as vitamin c).

Nutritional values per 100 mL

	lemon	Lemon-light	peach	Peach-light	mango	Mango-light
Energy	170kJ/ 40 kcal	5/<17kJ/1<4kcal	170kJ/40 kcal	5/<17kJ/1<4 kcal	170kJ/40kcal	5/<17kJ/1<4 kcal
Carbohydrates	10 g	0.1/<0.5 g	10 g	0.1/<0.5g	10 g	0.1/<0.5 g
Protein	0 g	0 g	0 g	0 g	0 g	0 g
Fat	0 g	0 g	0 g	0 g	0 g	0 g
Fibre	0 g	0 g	0 g	0 g	0 g	0 g
Sodium	<0.005 g	<0.005 g	<0,005 g	0.01 g	<0.005g	0,01 g

All drinks can be stored at room temperature and have a shelf life of 12 months. The drinks will be analyzed for sugar and artificial sweeteners content by an outside laboratory. Development and testing of these products according to our specifications is in progress. The drinks will be packaged and coded in such a way that neither the children, nor the parents and teachers, nor the investigators will know which treatment a child receives.

5.2 Sweeteners

In the study drinks, two artificial sweeteners are used: acesulfame K and sucralose. The use of these sweeteners is in strict accordance with government regulations (Voedingscentrum, European Commission's Scientific Committee on Food, European Food Safety Authority (EFSA)). Sucralose is the common name for a high-intensity sweetener made from sugar. Sucralose is 600 times sweeter than sugar. It does not break down in the body and it is non-caloric. Sucralose has been approved by the Joint FAO/WHO Expert Committee on Food Additives (JECFA) (1990) and by the Scientific Committee on Food (SCF) of the European Commission (2000) – now the European Food Safety Authority (EFSA).
Sucralose is an authorised sweetener in the *European Parliament and Council Directive 94/35/EC as amended by Directive 2003/115/EC*. Sucralose is currently approved for use in foodstuffs in more than 50 countries around the world, including the USA, Canada, Australia, Japan, China and Russia. In August 1999 the American Food & Drug Administration (FDA) published their approval of sucralose as a "general purpose sweetener in foods". This means that sucralose can be used in any food at GMP (Good Manufacturing Practice) levels in the USA. SAFETY: Extensive studies have been conducted to support the safety of sucralose. The results of these studies demonstrate that it is safe for human consumption.
The Acceptable Daily Intake (ADI) for sucralose was set at 15 mg/kg body weight by JECFA in 1990 and by the SCF in 2000.
In this study the amount of sucralose for a child of 15 kg (minimal weight for 5 year old children) can be calculated as follows: 250 ml light study drink contains 39 mg of sucralose. For a child weighing 15 kg the daily intake is 2.6 mg/kg. This is far below the ADI of 15

mg/kg. When children get heavier (older) the daily intake per kg body weight will decrease. Also acesulfame K is considered to be a safe artificial sweetener. The European Commission's Scientific Committee on Food (SCF) set the ADI of acesulfame K on 9 mg/kg/day. In this study the amounts of acesulfame K for a child of 15 kg (minimal weight for 5 year old children) can be calculated as follows: 250 ml light study drink contains 14 mg of Acesulfame K. For a child weighing 15 kg the daily intake is 1 mg/kg. This is far below the ADI of 9 mg/kg. When children get heavier (older) the daily intake per kg body weight will decrease.

Packaging of the drinks

All cans will be printed in an attractive way for children containing general information such as name and quantity. Ingredients and nutritional values will not be printed on the drinks because the pilot study/ main trial are blinded. Before the pilot study/ main trial starts parents will receive nutritional information about all drinks so they know what his/her child could be drinking. Besides printing the drinks, all cans will be labelled with stickers with the name of the child. Labelling the drinks will be done by an independent person who is not further involved in the pilot study/ main trial directly.

5.3 Use of co-intervention (if applicable)

Not applicable

5.4 Escape medication (if applicable)

Not applicable

6. INVESTIGATIONAL MEDICINAL PRODUCT

Not applicable

7. METHODS

7.1 Study parameters/endpoints

7.1.01 Main study parameter/endpoint

Primary endpoints will be measured at the beginning and at 6, 12 and 18 months of the trial: body mass index (corrected for age) and fat mass as estimated from waist circumference (cm), skin fold thickness (mm) and bioelectrical impedance.
Skin fold thickness will be measured at the biceps and triceps. Skin fold thickness, waist circumference and bioelectrical impedance are good indices of fat mass in children. We chose these measures also because they can be easily done at the schools.

7.1.02 Secondary study parameters/endpoints

Secondary endpoint is the energy intake at lunch time shortly after the morning break when the children have consumed the study drinks. This will be measured twice during the main trial: at the start (month 0) and at the end of the main trial (month 18). Parents will be informed that they do not have to give a lunch meal to school on that specific day. On that day children will drink the study drinks during the morning break. During lunch time, children will be offered a lunch from which they may eat ad libitum. During this lunch children are not allowed to eat food brought from home. After the lunch researchers will make notes of what each child has exactly eaten during the lunch and energy intake will be calculated. In this way we can compare energy-intake of children who consume sugary beverages with children who consume the low sugar alternatives.

7.1.03 Other study parameters

Not applicable

7.2 Randomisation, blinding and treatment allocation

Randomization

Before randomizing children in two groups, participants will stratified based on age, gender, BMI, school and class. Subsequently, subjects will be randomly assigned to one of the two groups by a computer generated randomization.

Blinding and treatment allocation

The treatments will be double blind. This means that drinks will be packaged and coded in such a way that neither the children, nor their parents or teachers, nor the investigators will know which treatment a child receives. The packaging and coding will be done by a person not further involved in the trial. The code will be kept in a sealed envelop. The principal investigator will perform unblinding of the treatment allocation after the trial has

ended and analyses are complete. The codes can be broken if serious adverse health effects would occur.

7.3 Study procedures

Informed consent of the parents will be obtained for the pilot study/ main trial separately and then parents fill out a questionnaire. Based on the questionnaire we will decide who can participate in the pilot study/ main trial.
The main trial will start with a run in period of 2 weeks in which all children receive the sugary drinks. The run in period is meant to make children, parents and the school familiar with the study procedures. During this run in period, baseline measurements (measurement 1) will be done. Following the run in period the study period of 18 months will start.

Drinks at school
During the study children consume the study drinks daily during the morning break at school under supervision of the teacher. In this way the teacher can check whether the children ingest their assigned drink. Empty packages will be collected and counted to check compliance
Drinks at home
During weekends and holidays parents will be asked to ensure that children consume the drinks daily as a replacement of the drinks that they normally receive. Also parents will be asked to collect the empty packages and take them to school so the teacher can register if children consumed the drinks at home.

Several actions will be taken for the children to ensure compliance and limit drop out during the pilot study/ main trial, such as the opportunity for children to collect stickers, news letter every few months, fancy website, games, puzzles etc.

Measurements during the study
During the pilot study children will be measured twice and during the trial four times. Measurements will be done by an experienced, trained research team. This research team will receive training so they know all about how to measure length, weight, skin folds, waist circumference, bioelectrical impedance and food intake during lunch. Measurements will be done in consultation with the school management for timing and locations of the measurements.. Length will be measured with a portable wall-mounted stadio meter. Body weight will be measured with a electronic calibrated scale, to the nearest 1 gr. Skin folds will be measured at the triceps and the biceps using a skin fold calliper. Skin fold measurements will only be done by two people, the PhD student of this trial: Janne de Ruyter and her

assistant. Waist circumference will be measured by placing a centimetre around the trunk right in between the lowest rib and the iliac crest. Also a bioelectrical impedance analysis (BIA) will be done for each child. BIA determines the electrical impedance, or opposition to the flow of an electric current through body tissues which can then be used to calculate an estimate of total body water. Total body water can be used to estimate fat-free body mass and, by difference with body weight, body fat. Food intake at lunch will be measured through offering a lunch at school from which the participating children can eat ad libitum (for more information, see paragraph 7.1.02).

7.4 Withdrawal of individual subjects

Subjects or their parents can discontinue the pilot study/ main trial at any moment without the obligation tot state the reason for discontinuation. Subjects may be withdrawn from the pilot study/ main trial by the principal investigator in case of reported serious adverse events or other medical or psychological or social event as evaluated by the independent physician and discussed with the principal investigator.

7.4.01Specific criteria for withdrawal

Not applicable

7.5 Replacement of individual subjects after withdrawal

The trial will start with 600 subjects to account for possible drop-outs. It is assumed that at least the necessary (with regard to the statistical power) 500 subjects will complete the trial.

7.6 Follow-up of subjects withdrawn from treatment

Each subject who does not complete the pilot study/ main trial for any reason should be contacted and should have a post-study screen if requested by the subject, researcher or independent physician.

7.7 Premature termination of the study

There are no health risks related to premature termination of the pilot study/ main trial. Children can be excluded from the pilot study/ main trial if they themselves, parents or the researcher want them to do so. In case of premature termination, children will be contacted for a post-study screen.

8. SAFETY REPORTING

8.1 Section 10 WMO event

In accordance to section 10, subsection 1, of the WMO, the investigator will inform the subjects and the reviewing accredited METC if anything occurs, on the basis of which it appears that the disadvantages of participation may be significantly greater than was foreseen in the research proposal. The study will be suspended pending further review by the accredited METC, except insofar as suspension would jeopardise the subjects' health. The investigator will take care that all subjects are kept informed.

8.2 Adverse and serious adverse events

Adverse events are defined as any undesirable experience occurring to a subject during a clinical trial, whether or not considered related to the investigational drug. All adverse events reported spontaneously by the subject or observed by the investigator or his staff will be recorded.

A serious adverse event is any untoward medical occurrence or effect that at any dose results in death;

- is life threatening (at the time of the event);
- requires hospitalisation or prolongation of existing inpatients' hospitalisation;
- results in persistent or significant disability or incapacity;
- is a congenital anomaly or birth defect;
- is a new event of the trial likely to affect the safety of the subjects, such as an unexpected outcome of an adverse reaction, lack of efficacy of an IMP used for the treatment of a life threatening disease, major safety finding from a newly completed animal study, etc.

All SAEs will be reported to the accredited METC that approved the protocol, according to the requirements of that METC.

8.2.01 Suspected unexpected serious adverse reactions (SUSAR)

Not applicable

8.2.02 Annual safety report

Not applicable

8.3 Follow-up of adverse events

All adverse events will be followed until they have abated, or until a stable situation has been reached. Depending on the event, follow up may require additional tests or medical procedures as indicated, and/or referral to the general physician or a medical specialist.

8.4 Data Safety Monitoring Board (DSMB)

Not applicable

9. STATISTICAL ANALYSIS

9.1 Descriptive statistics

This trial will be a quantitative study. The primary analysis of the effect of sugary drinks will be performed on an intention-to-treat basis of all children as randomized. Groups will be stratified on gender, age, BMI and school. Differences in the outcome variables between the 2 intervention groups will be analyzed with linear regression analysis, with correction for potential confounders. Analyses will be performed with SPSS.

9.2 Univariate analysis

9.3 Multivariate analysis

9.4 Interim analysis

Not applicable

10 ETHICAL CONSIDERATIONS

10.1 Regulation statement

<*In this section it can be stated that the study will be conducted according to the principles of the Declaration of Helsinki (*59th WMA General Assembly, Seoul, October 2008) *and in accordance with the Medical Research Involving Human Subjects Act (WMO)*

10.2 Recruitment and consent

Children will be recruited through primary schools. These schools will be recruited through collaborations that already exist between our institute and primary schools. Schools will be carefully selected mainly based on the willingness of school board and teachers to participate in the execution of the pilot study/ main trial. If schools can not guarantee involvement in the execution of the pilot study/ main trial, they can not participate. If schools are willing to participate in the pilot study/ main trial, parents will be informed about the pilot study/ main trial orally and in writing by the researchers. The researchers will send parents an information letter (containing all important information, including contact information) and an informed consent form. Soon after that a parent evening will be organised to inform parents about the exact procedures during the pilot study/ main trial. Parents may give the signed informed consent form to the teacher of the child or to the researchers after the parent evening. They can also take two days of consideration after the parent evening before they return the informed consent to the teacher. A deadline for returning the informed consent form to the teacher will be mentioned in the information letter.

After the pilot study we will also organise a parent evening to evaluate the pilot. Parents will be asked to fill in a questionnaire so the research team knows what should be improved for the trial. After the trial parents will receive a report about the findings of this trial.

During the pilot study/ main trial the research team is always available for questions.

10.3 Objection by minors or incapacitated subjects (if applicable)

The parents should sign an informed consent form to agree that the child can participate in this pilot study/ main trial. We will inform parents that they have to ask the child whether he/she is willing to participate. If the child is not willing to participate he or she can not participate. If the child refuses to drink the study drinks the child can be withdrawn from the study

10.4 Benefits and risks assessment, group relatedness

The risk associated with participation in this pilot study/ main trial is considered negligible. All measurements are non-invasive measurements (weight, length, waist circumference, skin folds and bioelectrical impedance) and enhance common procedures with minimal burden involved. The sugary drinks used in the pilot study/ main trial are not different from regular sugary drinks available on the market for children. The study drinks will replace the sugary drinks that children already consumed before the pilot study/ main trial started. Therefore intake of energy and sugars from drinks will not change for the group consuming the sugary study drinks. The light study drink contains artificial sweeteners. The group that will receive the low-caloric study drink daily will thus decrease the daily intake of sugars from beverages because they replace the habitual sugary drink by the low-caloric study drink. The use of artificial sweeteners in the low-caloric study drink is in strict accordance with government regulations (http://www.voedingscentrum.nl), so no risk is associated to consumption of these drinks.

Since it is unethical to encourage children to drink sugary drinks, only subjects who already consume sugar containing beverages daily will participate in this pilot study/ main trial. All children will replace their habitual drinks with the study drinks, without a need to change their drinking habits. There will not be direct medical benefits for the participants. Eventually the results of this trial might lead to effective interventions that contribute to the prevention of overweight and obesity in children.

<Please give a justification of the proposed study.>

10.5 Compensation for injury

An insurance is available for all subjects participating in this study which is in accordance with the legal requirements in the Netherlands (Article 7 WMO and Medical Research (Human Subjects) Compulsory Insurance Decree of 23 June 2003. This insurance provides cover for damage to research subjects through injury or death caused by the study.

1. € 450.000,-- (i.e. four hundred and fifty thousand Euro) for death or injury for each subject who participates in the Research;
2. € 3.500.000,-- (i.e. three million five hundred thousand Euro) for death or injury for all subjects who participate in the Research;
3. € 5.000.000,-- (i.e. five million Euro) for the total damage incurred by the organisation for all damage disclosed by scientific research for the Sponsor as ‘verrichter’ in the meaning of said Act in each year of insurance coverage.

Contact information of the insurance company: Onderlinge Waarborgmaatschappij Centramed b.a., Postbus 191, 2270 AD Voorburg. The insurance company and insurance meet the requirements of the decision for the obligation of insurance for medical research with human subjects (Staatsblad 2003, 266). The subjects participating will be informed about this insurance.

10.6 Incentives

Not applicable

11. ADMINISTRATIVE ASPECTS AND PUBLICATION

11.1 Handling and storage of data and documents

Data will be handled confidentially and anonymously. If it is necessary to trace data to an individual subject, a subject identification code list will be used to link the data to the subject. The code will not be based on the patient initials and birth-date. The key to the code will be safeguarded by the principal investigator and independent randomizer. Documents related to the pilot study/ main trial will be kept in a locked room and are only accessible to the study investigator. The handling of personal data will comply with the Dutch Personal Data Protection Act.

11.2 Amendments

Amendments are changes made to the research after a favourable opinion by the accredited METC has been given. All amendments will be notified to the METC that gave a favourable opinion. Non-substantial amendments will not be notified to the accredited METC and the competent authority, but will be recorded and filed by the investigator. Examples of non-substantial amendments are typing errors and administrative changes like changes in names, telephone numbers and other contact details of involved persons mentioned in the submitted study documentation.

11.3 Annual progress report

The investigator will submit a summary of the progress of the trial to the accredited METC once a year. Information will be provided on the date of inclusion of the first subject, numbers of subjects included and numbers of subjects that have completed the trial, serious adverse events/ serious adverse reactions, other problems, and amendments.

11.4 End of study report

The investigator will notify the accredited METC of the end of the study within a period of 8 weeks. The end of the study is defined as the last measurement in the last participant .
In case the study is ended prematurely, the investigator will notify the accredited METC, including the reasons for the premature termination. Within one year after the end of the trial, the investigator/sponsor will submit a final study report with the results of the trial, including any publications/abstracts of the trial, to the accredited METC.

11.5 Public disclosure and publication policy

The results of this trial will be published in international peer-reviewed scientific journals and in a PhD thesis as soon as possible no matter what the outcome is.

REFERENCES

1. Schokker DF, et al.:Prevalence of overweight and obesity in the Netherlands. *Obes Rev* 8:101-108, 2007
2. Magarey AM, et al.:Predicting obesity in early adulthood from childhood and parental obesity. *Int J Obes Relat Metab Disord* 27:505-513, 2003
3. Garnett SP, et al.:Body mass index and waist circumference in midchildhood and adverse cardiovascular disease risk clustering in adolescence. *Am J Clin Nutr* 86:549-555, 2007
4. Hannon TS, et al.:Childhood obesity and type 2 diabetes mellitus. *Pediatrics* 116:473-480, 2005
5. Baan CA, Poos MJJC: Diabetes mellitus, omvang van het probleem. *In: Volksgezondheid Toekomst Verkenning, Nationaal Kompas Volksgezondheid.* Bilthoven, RIVM, 2005
6. Must A, Strauss RS:Risks and consequences of childhood and adolescent obesity. *Int J Obes Relat Metab Disord* 23 Suppl 2:S2-11, 1999
7. Latner JD, Stunkard AJ:Getting worse: the stigmatization of obese children. *Obes Res* 11:452-456, 2003
8. Janssen I, et al.:Associations between overweight and obesity with bullying behaviors in school-aged children. *Pediatrics* 113:1187-1194, 2004
9. van den Hurk K, et al.: Prevalentie van overgewicht en obesitas bij jeugdigen 4-15 jaar in de periode 2002-2004. Leiden, TNO Kwaliteit van leven, 2006
10. Polder JJ, et al.:Kosten van Ziekten in Nederland - De zorgeuro ontrafeld *Themarapport van de Volksgezondheid Toekomst Verkenning 2002. RIVM-rapport nr. 270751005. Bilthoven: RIVM*, 2002
11. Visscher TL, Seidell JC:The public health impact of obesity. *Annu Rev Public Health* 22:355-375, 2001
12. Wang LY, et al.:Economic analysis of a school-based obesity prevention program. *Obes Res* 11:1313-1324, 2003
13. Malik VS, et al.:Intake of sugar-sweetened beverages and weight gain: a systematic review. *Am J Clin Nutr* 84:274-288, 2006
14. DiMeglio DP, Mattes RD:Liquid versus solid carbohydrate: effects on food intake and body weight. *Int J Obes Relat Metab Disord* 24:794-800, 2000
15. Raben A, et al.:Sucrose compared with artificial sweeteners: different effects on ad libitum food intake and body weight after 10 wk of supplementation in overweight subjects. *Am J Clin Nutr* 76:721-729, 2002
16. Tordoff MG, Alleva AM:Effect of drinking soda sweetened with aspartame or high-fructose corn syrup on food intake and body weight. *Am J Clin Nutr* 51:963-969, 1990
17. James J, et al.:Preventing childhood obesity by reducing consumption of carbonated drinks: cluster randomised controlled trial. *Bmj* 328:1237, 2004
18. Ebbeling CB, et al.:Effects of decreasing sugar-sweetened beverage consumption on body weight in adolescents: a randomized, controlled pilot study. *Pediatrics* 117:673-680, 2006
19. Zo eet nederland 1998. Resultaten van de voedselconsumptiepeiling 1998. Den Haag, Voedingscentrum, 1998

20. Wells JC, Fewtrell MS: Measuring body composition. Arch Dis Child 91:612-617, 2006
21. Wang YC, Gortmaker SL, Sobol AM, Kuntz KM: Estimating the energy gap among US children: a
counterfactual approach. Pediatrics 118:e1721-1733, 2006
22. de la Hunty A, Gibson S, Ashwell M: A review of the effectiveness of aspartame in helping with weight control. Nutrition Bulletin 31:115-128, 2006
23. Linda van Rooij-van den Bos; Erik J.M. Konings1; Peter Heida; Ingrid C.M. van Hamersveld; Jacqueline van der Wielen; Martin Kooijman. Sacharine, Aspartaam, Acesulfaam-K en Cyclamaat in levensmiddelen Gehaltebepaling en inname door de Nederlandse populatie. Voedsel en Waren Autoriteit / Keuringsdienst van Waren, regio Zuid Voedsel en Waren Autoriteit / Keuringsdienst van Waren, regio Noord. Projectnummer: ZD 03 K12

Amendement 1:

VU medisch centrum
METc, t.a.v. Mevrouw W. vd Voet
PK 6 Z 202
De Boelelaan 1118
Postbus 7075
1007 MB Amsterdam

18 augustus 2009

Betreft: update METc protocol 09/034

Geachte mevrouw van der Voet,

Bijgaand willen wij u informeren over enkele kleine aanpassingen van het studieprotocol 09/034 *'A double-blind randomized trial of the efficacy of replacing sugary drinks by low-sugar alternatives on body weight and fat mass in school children'.* Bijgaand sturen wij u de laatste versie van de informatiebrief voor de ouders en de vragenlijst voor de
DRINK-studie. De aanpassingen in het kort:

- E1 informatiebrief ouders en E2 toestemmingsformulier: de tekst hebben wij ingekort en wij
hebben de brief samengevoegd met het toestemmingsformulier zodat het geheel als folder (A-3 formaat) gedrukt kan worden. Inhoudelijk is de informatie in de informatiebrief gelijk gebleven en alle benodigde informatie wordt verstekt.

- F1 vragenlijst DRINK-studie: we hebben 4 vragen geschrapt (vraag 6, 9, 11, 12 en 17 oude
versie) en wij hebben 3 vragen toegevoegd (vraag 6, 7 en 8 nieuwe versie: *6. Eet uw kind iets
tijdens de eerste ochtendpauze op school? 7. Eet uw kind tijdens een normale schooldag de
lunch op school of thuis? 8.Wat eet en drinkt uw kind gemiddeld tijdens de lunch?*)
Daarnaast willen wij u informeren dat wij een secundaire uitkomstmaat hebben toegevoegd, namelijk'tandgezondheid'. Aan het eind van de studie zullen wij dit meten d.m.v. twee vragen aan de ouders betreffende de behandelingen die hebben plaatsgevonden tijdens de tandartsbezoeken gedurende de DRINK-studie (nl. aantal vullingen en aantal tanden/kiezen verwijderd door cariës). Als laatste willen wij u informeren dat de startdatum van het onderzoek is: 29 september 2009. Wij hopen u hiermee voldoende geïnformeerd te hebben.

Wij zijn ten alle tijde bereid vragen te beantwoorden.

Met vriendelijke groet,
Prof. dr. Martijn B. Katan

Amendement 2:

VU medisch centrum
METc, t.a.v. Mevrouw W. vd Voet
PK 6 Z 202
De Boelelaan 1118
Postbus 7075
1007 MB Amsterdam

vrije Universiteit amsterdam

12 november 2009

Betreft: update METc protocol 09/034

Geachte mevrouw van der Voet,

Bijgaand sturen wij u het aangepaste ABR-formulier zoals verzocht in uw e-mail van 22 september jl.
Het betreft het studieprotocol 09/034 *'A double-blind randomized trial of the efficacy of replacing sugary drinks by low-sugar alternatives on body weight and fat mass in school children'.*

Wij hebben nav onze brief dd 18 augustus 2009 de secundaire maat 'tandgezondheid' toegevoegd.

Daarnaast hebben wij nog 4 aanpassingen gemaakt:

1) Wij hebben een secundaire maat toegevoegd, nl 'sensorische evaluatie van de dranken'.
2) De meting van de voedingsinname bij de lunch zal alleen aan het einde van de studie plaatsvinden en niet aan het begin.
3) Wij vragen kinderen om een kleine beetje urine te verzamelen bij elke meting. In de urine wordt de hoeveelheid sucralose gemeten (zoetstof die in de dranken zit). De hoeveelheid sucralose in urine zal worden gebruikt als een compliance marker voor consumptie van de dranken.
4) Wij hebben de metingen 'middelomtrek, huidplooien, lengte en bioimpedantie' verplaatst van primaire uitkomstmaten naar secundaire uitkomstmaten.

Bijgaand de tekst zoals die nu in het ABR formulier is aangepast:

Paragraaf E4:

-non-invasieve metingen 4 maal gedurende de studie: lichaamsgewicht, lengte, middelomtrek, huidplooien, bioimpedantie.
-gedurende 1,5 jaar elke dag een drankje te drinken die wij voor dit onderzoek hebben geselecteerd. Dit drankje is een vervanging van de drank die normaalgesproken gedronken wordt.
-5 maal een korte vragenlijst (4 vragen) betreft sensorische evaluatie van de dranken

-1 maal korte vragenlijst (2 vragen) aan de ouders betreft tandgezondheid van de kinderen
-4 maal kleine urineverzameling ten behoeve van meting van sucralose in urine als compliance marker

Paragraaf K1 'Secundaire onderzoeksvariabelen/uitkomstmaten' en K2 (Engelse vertaling):

De secundaire uitkomstmaten van dit onderzoek zijn:
- Middelomtrek, 4 huidplooien, lichaamslengte en bioimpedantie. Deze uitkomstmaten zullen vier keer worden gemeten gedurende de interventieperiode van 1, 5 jaar (aan het begin, na 6, 12 maanden en aan het einde van de trial).
- Tandgezondheid. Aan het eind van de studie zullen wij dit meten d.m.v. twee vragen aan de ouders betreffende de behandelingen die hebben plaatsgevonden tijdens de tandartsbezoeken gedurende de DRINK-studie (nl. aantal vullingen en aantal tanden/kiezen verwijderd door cariës).
- Sensorische evaluatie van de dranken gedurende de studie. Deze uitkomstmaat bestaat uit 4 vragen: 1) Hoeveel zin heb je in dit drankje? 2) Hoe vol zit je? 3) Wat eet je bij het drankje? 4) Hoe lekker vind je het drankje. De antwoorden van vragen 1, 2 en 4 worden door kinderen aangegeven op een 5-puntsschaal, vraag 3 is een open vraag. De vragenlijst is gevalideerd bij kinderen. De vragenlijst wordt 5 maal afgenomen gedurende de studie (run in, week 1, 6, 12 en 18 maanden)
- Voedingsinname tijdens de lunch, kort na de eerste pauze waarin kinderen het studiedrankje hebben gedronken. Deze uitkomstmaat zal aan het einde van de studie worden gemeten.
- Hoeveelheid sucralose in de urine, dit wordt gebruikt als compliance marker (therapietrouw).

Wij hopen u hiermee voldoende geïnformeerd te hebben.
Wij zijn ten alle tijde bereid vragen te beantwoorden.

Met vriendelijke groet,

Prof. dr. Martijn B. Katan

Bijlage:

herziene versie ABR formulier

Amendement 3:

VU medisch centrum
METc, t.a.v. Mevrouw W. vd Voet
PK 6 Z 202
De Boelelaan 1118
Postbus 7075
1007 MB Amsterdam

25 februari 2010

Betreft: update METc protocol 09/034

Geachte mevrouw van der Voet,

Graag willen wij het volgende melden betreft het studieprotocol 09/034 *'A double-blind randomized trial of the efficacy of replacing sugary drinks by low-sugar alternatives on body weight and fat mass in school children'.* In deze studie vragen wij de kinderen om een kleine beetje urine te verzamelen bij elke meting. In de urine wordt de hoeveelheid sucralose gemeten (zoetstof die in de dranken zit). De hoeveelheid sucralose in urine zal worden gebruikt als een compliance marker voor consumptie van de dranken. Echter, het is nog onduidelijk op welke tijdstippen de urineverzameling kan plaatsvinden. Daarvoor hebben wij een stage project opgezet wat wordt uitgevoerd door 4e jaars masterstudenten Gezondheidswetenschappen (VU). Binnen het stageproject voeren de studenten een klein onderzoek uit bij een aantal vrijwilligers. Opzet studie: 10 gezonde volwassen vrijwilligers (ouder dan 18 jaar) drinken gedurende 5 dagen elke dag 1 blikje (250 ml) limonade met sucralose (conform het studieprotocol 09/034, dezelfde limonade wordt gebruikt als in het onderzoek). Zij verzamelen in totaal 7 keer urine:
1) dag 1 (net voordat ze de limonade gaan drinken) en op dag 5 op de volgende tijdstippen:
2) 9.30 u (net voordat de laatste keer limonade gedronken wordt)
3) 10.30 u
4) 11.30 u
5) 12.30 u
6) 13.30 u
7) 14.30 u

Alle deelnemers worden schriftelijk geïnformeerd over het onderzoek en tekenen een informed consent formulier. Aangezien alle activiteiten die binnen dit stageproject zullen plaatsvinden al eerder zijn goedgekeurd door de METc (protocol 09/034) en aangezien dit een stageproject voor studenten betreft, gaan we er vanuit dat dit project niet apart door de commissie hoeft te worden beoordeeld.

Wij hopen u hiermee voldoende geïnformeerd te hebben. Wij zijn te allen tijde bereid vragen te beantwoorden.

Met vriendelijke groet,

Prof. dr. Martijn B. Katan
Vrije Universiteit
Afdeling gezondheidswetenschappen
Tel 020-5982610; e-mail: martijn.katan@falw.vu.nl
